# Study on secondary metabolites of endophytic fungus *Diaporthe* sp. AC1 induced by tryptophan analogs

**DOI:** 10.3389/fmicb.2023.1254609

**Published:** 2023-10-09

**Authors:** Shikai Zhang, Qing Xu, Changbo Ji, Xiaoyu Han, Yang Zhou, Chao Liang, Linran Ma, Weijian Sun, Yanling Li, Zhengyou Yang, Fengchun Zhao, Yuan Tian

**Affiliations:** ^1^Department of Microbiology, College of Life Science, Key Laboratory for Agriculture Microbiology, Shandong Agricultural University, Taian, China; ^2^College of Life Science, Shandong First Medical University & Shandong Academy of Medical Sciences, Taian, China

**Keywords:** *Diaporthe*, endophytic fungi, 1-methyl-L-tryptophan, secondary metabolites, biological activity

## Abstract

Small molecule-induced fermentation of the endophytic fungus *Diaporthe* sp. AC1 originated from *Artemisia argyi* was executed to investigate its secondary metabolites. It was fermented in a culture medium containing 5-hydroxytryptophan (5-HTP), 1-methyl-L-tryptophan (1-MT), and tryptamine (TA), respectively. The antibacterial activities of crude extracts against pathogenic bacteria and pathogenic fungi were determined by using the Oxford cup method, while the cytotoxicity of crude extracts against cancer cells was determined by using the MTT method. The results showed that the secondary metabolites of *Diaporthe* sp. AC1 induced by 1-MT exhibited optimal antibacterial activity and tumor cytotoxicity. The induction conditions of 1-MT were optimized, and the antibacterial activities and tumor cytotoxicity of crude extracts under different induction conditions were investigated. As indicated, the optimal moment for 1-MT addition was before inoculation and its optimal concentration was 0.25 mM. Under these conditions, *Diaporthe* sp. AC1 was fermented and approximately 12 g of crude extracts was obtained. The crude extracts were then separated and purified to acquire nine monomer compounds, including three new compounds (**1**–**3**) and six known compounds (**4**–**9**). The antibacterial activities of the compounds against pathogenic bacteria and pathogenic fungi were investigated by using the microdilution method, while their cytotoxicity against cancer cells was analyzed by using the MTT method. The results demonstrated that Compound **1** exhibited moderate antibacterial activities against *Verticillium dahlia*, *Fusarium graminearum,* and *Botrytis cinerea*, as well as a low inhibitory activity against *Listeria monocytogenes*. Nevertheless, Compound **1** showed significant cytotoxicity against five cancer cells, with IC_50_ ranging from 12.26 to 52.52 μM. Compounds **2** and **3** exhibited negligible biological activity, while other compounds showed detectable inhibitory activities against pathogenic bacteria and cancer cells.

## 1. Introduction

Endophytic fungi are characterized by rich diversity of species (Gokhale et al., 2017) and capability to generate compounds with pharmaceutical and commercial values (Deshmukh et al., 2018; Gao et al., 2018), thus being regarded as a goldmine of secondary metabolites (Uzma et al., 2018; White et al., 2019). Recent studies of the whole fungal genomes have demonstrated the presence of various silent gene clusters, indicating that a large proportion of new compounds remain to be identified (Hautbergue et al., 2018). Therefore, maximized activation of silent gene clusters has been playing a key role in efficient utilization of fungal resources in the post-genomic era.

One Strain Many Compounds (OSMAC) strategy is a facile and effective strategy to identify novel active metabolites (Liu et al., 2017). By tuning the medium composition (Ranuka et al., 2014) and culture conditions (Siridechakorn et al., 2017), co-culturing with other strains (Wang et al., 2022), and adding small molecule inducers (Gakuubi et al., 2022; Li et al., 2022), the metabolic pathway of secondary metabolites can be adjusted to identify novel secondary metabolites (Pinedo-Rivilla et al., 2022). Specifically, the addition of small molecule inducers leads to significant increase in the variety of secondary metabolites. It has been demonstrated that the addition of tryptophan analog 1-methyl-L-tryptophan (1-MT) into the culture of *Chaetomium globosum* 1C51 could activate cryptic gene and trigger the Pictet-Spengler reaction of 1-MT and flavipin to produce new indole alkaloids (Yan et al., 2014). Additionally, tryptamine, another analog of tryptophan, has been used as a “scavenger” to capture the biosynthetic intermediates of *Diaporthe* sp., resulting in the identification of novel alkaloids originated from tryptamine-exposed fungal culture (Chen et al., 2021).

*Diaporthe* is typically isolated as endophytic fungi and secondary metabolites produced by this genus have been thoroughly investigated (Chepkirui and Stadler, 2017). The endophytic fungus *Diaporthe* sp. AC1 derived from *Artemisia argyi* can produce various active natural products, including phomopsolides, pyranones, and indole alkaloids (Gu et al., 2022). The structural diversities and biological activities of the compounds encouraged further study on the use of *Diaporthe* sp. AC1 for the production of new metabolites by the “OSMAC” technique. Since metabolites production of endophytic fungi can be influenced by adding small molecule inducers as mentioned earlier, three analogs of tryptophan [1-MT, tryptamine, and 5-hydroxytryptophan (5-HTP)] were added into the fermented culture of this strain, with the aim of generation of new fungal metabolites. Herein, 1-MT was screened as the optimal inducer, and three new compounds (**1**–**3**) as well as six known ones were produced by the endophytic fungus *Diaporthe* sp. AC1 after adding 1-MT into media for fungal cultivation.

## 2. Materials and methods

### 2.1. Chemicals and reagents

The media used for the experiments included the potato dextrose agar (PDA) medium (200 g of potato, 20 g of dextrose, and 15 g of agar in 1000 mL of deionized water), the malt extract (ME) liquid medium (20 g of raw malt, 20 of g sucrose, and 1 g of peptone in 1000 mL of deionized water, pH = 7.2), and the Luria-Bertani (LB) agar medium (10 g of tryptone, 5 g of yeast extract, 10 g of NaCl, and 15 g of agar).

The organic solvent used for chromatographic separation was purchased from Tianjin Kaitong Chemical Reagent Co., Ltd., China. The silica gel (200–300 mesh) used for column chromatography was purchased from Qingdao Ocean Chemical Co., Ltd., China. Sephadex LH-20 gel was purchased from GE Healthcare. Thin-layer chromatography silica gel plates (GF254) were purchased from Qingdao Ocean Chemical Co., Ltd., China. 5-HTP, 1-MT, and tryptamine (TA) were purchased from Shanghai Aladdin Biochemical Technology Co., Ltd., China.

### 2.2. Fungal and bacterial materials

*Diaporthe* sp. AC1 was isolated from *Artemisia argyi* and identified by following a previously reported protocol (Gu et al., 2022). The ITS sequence data of this fungal strain have been submitted to the GenBank under the accession number of OL589639.

Pathogenic bacteria *Staphylococcus aureus* ATCC25923, *Listeria monocytogenes* CGMCC1.10753, *Salmonella enteritidis* ATCC13076, *Pseudomonas aeruginosa* ATCC27853 and *Escherichia coli* ATCC8739, as well as pathogenic fungi *Candida albicans* ATCC10231 were purchased from Shanghai Bioresource Collection Center (SHBCC). Pathogenic fungi *Fusarium graminearum*, *F*. *moniliforme*, *Botrytis cinerea* and *Verticillium dahlia* were isolated, identified and preserved by our laboratory (Gu et al., 2022).

### 2.3. Screening of tryptophan analogs

*Diaporthe* sp. AC1 was inoculated on PDA medium and incubated at 28°C for 5 days. Five disks (diameter = 1 cm) were then inoculated in 100 mL of ME liquid medium and shaken under 180 rpm at 28°C for 2 days. Afterwards, 5-HTP, 1-MT, and TA were separately added to the fungal cultures at 4 × 12 h intervals until a final concentration of 1 mM (Yan et al., 2019). After that, the fermentation broths were incubated for another 3 days. Herein, a fermentation broth without tryptophan analogs was used as the control group.

Whole culture broths (including mycelia) were extracted with equal volume of ethyl acetate for two times. Meanwhile, organic solvents were evaporated under reduced pressure to obtain crude extracts. Additionally, the antibacterial activities and cytotoxicity of the crude extracts were evaluated.

### 2.4. Optimization of induction conditions

According to the methods described before (Yan et al., 2019) with some modification, the addition moment and concentration of the tryptophan analogs were optimized as follows: for Groups 1 and 2, the inducer was added simultaneously with mycelia before fermentation at a concentration of 1 and 0.25 mM, respectively; for Group 3, the inducer was added after two-day fermentation at a concentration of 1 mM; for Group 4, inducer was added to culture after two-day fermentation at 4 × 12 h intervals until a concentration of 1 mM was reached.

### 2.5. Preparation of crude extract

Under optimized induction conditions, *Diaporthe* sp. AC1 was fermented to obtain crude extracts. Briefly, *Diaporthe* sp. AC1 was inoculated in the PDA medium and incubated at 28°C for 5 days. Subsequently, five disks (diameter = 1 cm) were inoculated in 100 mL of ME liquid medium and shaken under 180 rpm at 28°C for 5 days to obtain the seed solution, which was then transferred to 2.5 L of ME medium for induced fermentation. Herein, 50 L of culture (200 bottles of 2.5 L medium) was fermented. After fermentation, the crude extract was extracted and concentrated by using the method described above. Additionally, antibacterial activities and cytotoxicity of the crude extracts were evaluated.

### 2.6. Antibacterial activity of crude extract

As described in our previous study (Gu et al., 2022), the antibacterial activities of the crude extracts against five pathogenic bacteria and five pathogenic fungi were investigated by using the Oxford cup method. Firstly, four Oxford cups were placed uniformly on top of water agar which was poured into the Petri dish beforehand. Then, 150 μL of pathogen suspension (1.0 ~ 5.0 × 10^7^ CFU/mL) and 15 mL of medium (LB agar medium for bacteria, PDA medium for fungi) were poured into the Petri dish. After solidification, the Oxford cups were removed, resulting in generation of four small wells in the upper medium.

In the experimental group, 50 μL of the crude extract solution (20 mg/mL, dissolved in methanol) was added to each well. Ampicillin sodium (20 mg/mL) and amphotericin B (20 mg/mL) were used as the positive control of antagonistic bacteria and fungi, respectively. Then, 50 μL of methanol was added to the negative control wells. The plates inoculated with bacteria were cultured at 37°C for 24 h, and those containing fungi were cultured at 28°C for 5~7 days. Additionally, the diameters of inhibition zones were measured.

### 2.7. Cytotoxicity of crude extract

The cytotoxicity of the crude extracts against five cancer cells (human hepatoma cell line Huh-7, human cervical cancer cell line Hela, human colorectal adenocarcinoma cell line HCT-15, human lung cancer cell line A549, human breast cancer cell line MDA-MB-231) was analyzed by using the MTT method (Mosmann, 1983). Specifically, cancer cells were added to a 96-well plate (4 × 10^4^ cell/mL in DMEM (Dulbecco’s Modified Eagle Medium) containing 10% FBS, 100 μL/well) and incubated at 37°C (7% CO_2_) for 20 h. Then, 100 μL of the cell culture media containing compounds of different concentrations (50, 25, 12.5, 6.25 μg/mL) was added into different wells; the well added with 100 μL of the cell culture medium with no compound was used as the negative control, while the blank control group contained culture medium but no cells. After 48 h, the plate was rinsed once with PBS buffer (200 μL/well) and MTT solution (1 mg/mL in DMEM containing 10% FBS, 100 μL/well) was added, followed by another four-hour incubation. After that, the solution was removed and DMSO (150 μL/well) was added to dissolve the formazan crystals generated during incubation. After 10 min of shaking, the absorbance was measured at 570 nm. Each group had three replicates. The inhibition rates of the compounds were calculated by:


$$\mathrm{Inhibition}\;\mathrm{rate}\;\left(\%\right)=\left(1-\frac{A_{1}-A_{0}}{A_{2}-A_{0}}\right)\times 100\%$$

where A_0_, A_1_, and A_2_ refer to the absorbance of the blank control, the experimental group, and the negative control, respectively.

### 2.8. Purification and characterization of compounds

**Figure 1:**
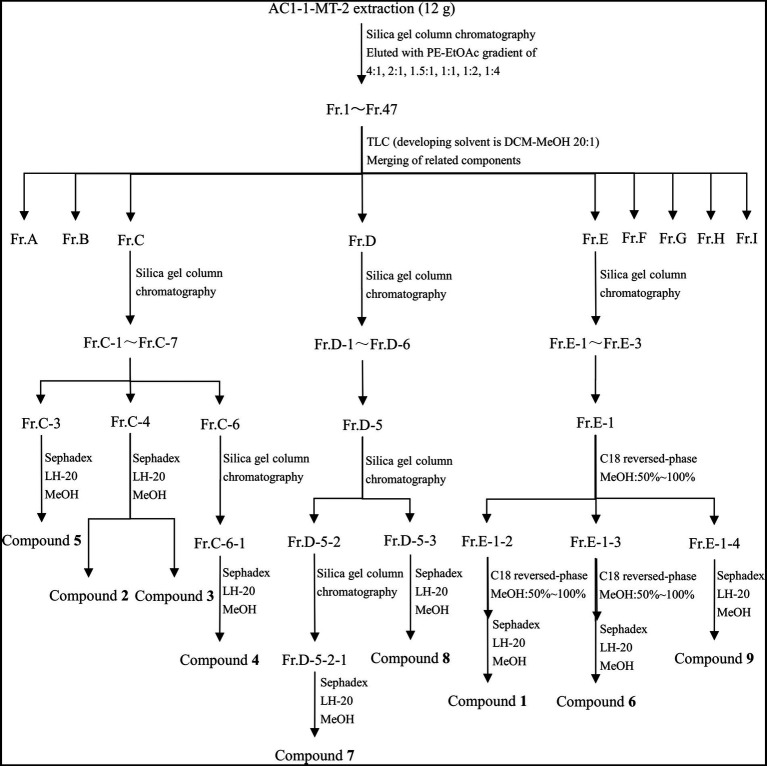
Purification process of secondary metabolites.

As shown in Figure 1, the crude extract was eluted by silica gel column chromatography with a gradient elution of PE-EtOAc (petroleum ether – ethyl acetate) to obtain 47 fractions (Fr. 1 ~ Fr. 47). Then, TLC was used to analyze each fraction and similar ones were combined, resulting in generation of seven subfractions (Fr.A ~ Fr.I). Among them, subfractions C, D, and E were further separated by using the C18 reverse-phase silica gel column with 50% ~ 100% methanol as the eluent. Additionally, the fractions were further separated by using the Sephadex LH-20 gel column.

The compounds were dissolved with CDCl_3_ or acetone-*d*_6_, and identified by nuclear magnetic resonance (NMR), high-resolution mass spectrometry (HR-MS), and/or two-dimensional nuclear magnetic resonance (2D-NMR). ^1^H NMR (600 MHz) and ^13^C NMR (151 MHz) spectra of the samples were obtained by using the NMR spectrometer (Bruker Avance, Germany) with tetramethylsilane (TMS) as the internal standard. Additionally, HR-MS spectra of the samples were obtained by using an AB SCIEX X500R QTOF mass spectrometer.

### 2.9. Antimicrobial activity of compounds

Preparation of bacterial suspension: Pathogenic bacteria were inoculated in LB liquid medium, and incubated at 37°C 200 rpm for 12 h. The bacterial culture was then diluted with LB liquid medium so that its OD_600_ value reached to 0.2. The culture was further diluted 300 times to obtain a bacterial suspension in which the concentration was 1 ~ 5 × 10^5^ CFU/mL.

Preparation of fungal suspension: Pathogenic fungi were incubated at 28°C on PDA medium for 3 ~ 7 days, the dishes were then rinsed with sterile water to prepare spore suspension. The spores were counted using the Hemocytometer, and the concentration of spores was diluted to 1 ~ 5 × 10^5^ CFU/mL with PDB medium.

Modified microdilution method (Zgoda and Porter, 2001) was used to evaluate the antimicrobial activities of the compounds against pathogenic bacteria and fungi. Firstly, 100 μL of the cell suspension (containing 1 ~ 5 × 10^5^ CFU/mL bacteria or fungal spores) was added to each well of a 96-well plate. Then, 100 μL of the culture medium (LB medium for bacteria and PDB medium for fungi) containing compounds of different concentrations (512, 256, 128, 64, 32 μg/mL) was added into each well. The well added with 100 μL of culture medium but no compound was used as the negative control, while the blank control contained culture medium but no pathogenic bacteria or compounds. Each group had three replicates. The 96-well plate inoculated with bacteria was incubated at 37°C for 24 h, while the plate containing fungi was incubated at 28°C for 48 h. The absorbance was measured at 600 nm. The inhibition rates of compounds were calculated by:


$$\mathrm{Inhibition}\;\mathrm{rate}\;\left(\%\right)=\left(1-\frac{A_{1}-A_{0}}{A_{2}-A_{0}}\right)\times 100\%$$

where A_0_, A_1_, and A_2_ refer to the absorbance of the blank control, the experimental group, and the negative control, respectively.

### 2.10. Cytotoxicity of compounds

The cytotoxicity of the compounds against five cancer cells was determined by using the MTT method as described above. The final concentrations of the compounds ranged from 200 to 12.5 μM with two-fold serial dilution.

## 3. Results and discussion

### 3.1. Screening of tryptophan analogs

As indicated by previous studies, tryptophan analogs 1-MT could change the metabolic pathway of *Chaetomium globosum* 1C51 and generate novel active compounds (Yan et al., 2019), and addition of tryptamine to *Diaporthe* sp. resulted in three novel alkaloids (Chen et al., 2021). In order to further explore the active metabolites of *Diaporthe* sp. AC1, an endophytic fungus from *Artemisia argyi* with antimicrobial potential, was fermented with three tryptophan analogs (5-HTP, 1-MT, and TA), respectively.

The results in Supplementary Table S1 revealed that the yields of crude extracts from fermentation broth added with tryptophan analogs were significantly higher than that of the control group (CK), indicating that addition of three amino acid analogs has positive impacts on the yield of fermentation products. The yield of fermentation products followed the order of TA (305.0 mg/L) > 5-HTP (296.5 mg/L) > MT (253.0 mg/L).

The antibacterial activities of the crude extracts are summarized in Table 1 and Supplementary Figure S1. Compared with those induced by no tryptophan analogs, crude extracts induced by 5-HTP exhibited enhanced antibacterial activity against *L. monocytogenes* and *S. aureus*, and those induced by TA exhibited enhanced inhibitory activity against *E. coli*, *S. enteritidis,* and *P. aeruginosa.* In particular, crude extracts induced by 1-MT exhibited optimized activity, as reflected by enhanced inhibitory activity against all of the five pathogenic bacteria involved.

**Table 1:** Antimicrobial effects of four kinds of crude extracts on pathogenic bacteria.

Number	Inhibition zone diameter (mm)				
	*L. monocytogenes*	*E. coli*	*S. enteritidis*	*S. aureus*	*P. aeruginosa*
CK	15.73 ± 0.21	11.73 ± 0.21	13.10 ± 0.20	17.13 ± 0.35	12.90 ± 0.30
5-HTP	18.17 ± 0.31	-	-	18.57 ± 0.21	12.23 ± 0.31
1-MT	20.03 ± 0.50	16.97 ± 0.21	20.27 ± 0.32	19.43 ± 0.21	16.70 ± 0.36
TA	14.20 ± 0.30	14.57 ± 0.31	17.77 ± 0.25	13.73 ± 0.21	13.93 ± 0.15
Ampicillin sodium	34.17 ± 0.35	31.93 ± 0.15	34.17 ± 0.31	34.07 ± 0.38	27.03 ± 0.42
MeOH	-	-	-	-	-

“-” no obvious antimicrobial effect. The outer diameter of the Oxford cup was 8 mm. The concentration tested was 20 mg/mL. CK, crude extract without tryptophan analogs; 5-HTP, crude extract added with 5-HTP; 1-MT, crude extract added with 1-MT; TA, crude extract added with TA. Three replicates were carried out for each test.

According to Table 2 and Supplementary Figure S2, the antifungal activities of crude extracts induced by 5-HTP and TA decreased compared with those of crude extracts induced by no tryptophan analogs. However, the crude extracts induced by 1-MT exhibited a slight increase in inhibitory activity against *C. albicans*, *F. moniliform*, and *B. cinerea.*

**Table 2:** Antimicrobial effects of four kinds of crude extracts on pathogenic fungi.

Number	Inhibition zone diameter (mm)				
	*C. albicans*	*V. dahliae*	*F. moniliforme*	*F. graminearum*	*B. cinerea*
CK	11.07 ± 0.15	13.50 ± 0.36	16.57 ± 0.68	15.80 ± 0.30	15.63 ± 0.61
5-HTP	-	10.67 ± 0.15	12.53 ± 0.40	13.60 ± 1.35	13.87 ± 0.35
1-MT	12.00 ± 0.26	12.83 ± 0.31	16.73 ± 0.47	12.43 ± 0.76	17.57 ± 0.35
TA	-	-	10.83 ± 0.31	15.67 ± 0.55	-
Amphotericin B	16.80 ± 0.30	18.77 ± 0.31	17.10 ± 0.30	14.67 ± 0.55	20.50 ± 1.40
MeOH	-	-	-	-	-

“-” no obvious antimicrobial effect. The outer diameter of the Oxford cup was 8 mm. The concentration tested was 20 mg/mL. CK, crude extract without tryptophan analogs; 5-HTP, crude extract added with 5-HTP; 1-MT, crude extract added with 1-MT; TA, crude extract added with TA. Three replicates were carried out for each test.

**Figure 2:**
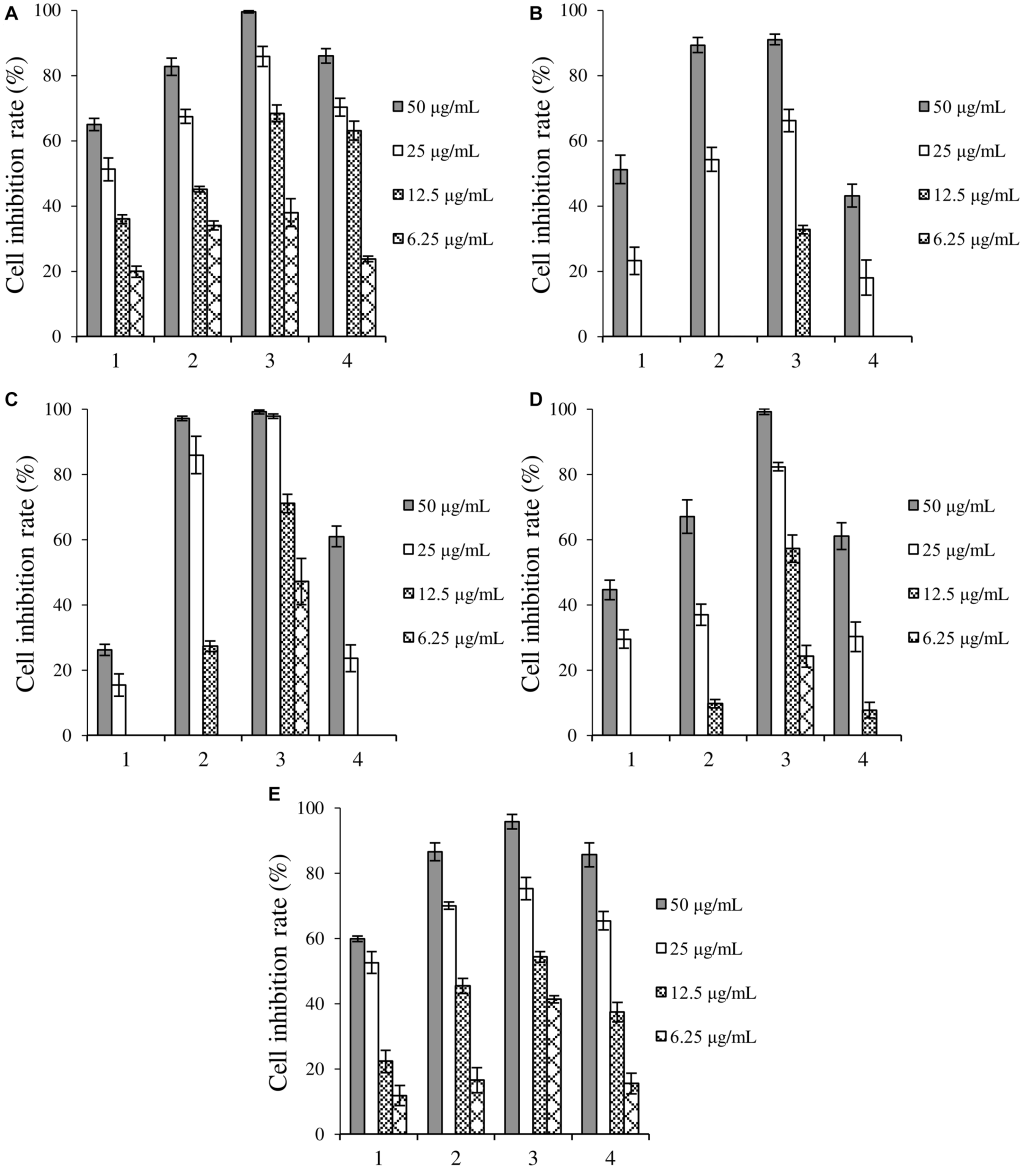
Inhibition rates of four kinds of crude extracts on cancer cells. **(A)** liver cancer cells Huh-7, **(B)** cervical cancer cell Hela, **(C)** colorectal adenocarcinoma cell HCT-15, **(D)** lung cancer cell A549, **(E)** breast cancer cells MDA-MB-231. 1: control, crude extract without tryptophan analogs, 2: crude extract added with 5-HTP, 3: crude extract added with 1-MT, 4: crude extract added with TA.

The cytotoxicity of crude extracts is summarized in Figure 2. All crude extracts involved in this study exhibited inhibitory activities against the five cancer cells in a dose-dependent manner. Compared with the control group, the crude extracts induced by 5-HTP and 1-MT exhibited enhanced cytotoxicity against the five cancer cells, while those induced by TA showed enhanced activity against Huh-7, HCT-15, A549, and MDA-MB-231. Additionally, crude extracts induced by 1-MT exhibited the optimal inhibitory activity, with inhibitory rates above 90 and 65% at concentrations of 50 and 25 μg/mL, respectively.

In summary, crude extracts induced by 1-MT exhibited the optimal antimicrobial and inhibitory performance toward cancer cells. Hence, 1-MT was employed as the inducer for subsequent experiments.

### 3.2. Optimization of induction conditions

The yields and characteristics of crude extracts induced by 1-MT are shown in Supplementary Table S2. 1-MT-1 and 1-MT-2 denoted crude extracts induced by 1 and 0.25 mM 1-MT, respectively; 1-MT-3 represented the crude extract generated by adding 1 mM 1-MT after two-day fermentation; 1-MT-4 indicated the crude extract generated by adding 1-MT after two-day fermentation at 4 × 12 h intervals until a concentration of 1 mM was reached. The yields of 1-MT-2, 1-MT-4, 1-MT-1, and 1-MT-3 were 251.0, 253.0, 242.0, and 240.0 mg/L, respectively.

The inhibition activities of the crude extracts fermented under four induction conditions against pathogenic bacteria and pathogenic fungi are shown in Tables 3, 4, and Supplementary Figures S3, S4. 1-MT-2 exhibited the optimal inhibition performance, with the diameters of inhibition zones of *L. monocytogenes*, *S. enteritidis*, *S. aureus*, *F. moniliforme*, and *B. cinerea* being above 20 mm. Moreover, its antibacterial activities against *E. coli*, *C. albicans*, *V. dahlia*, and *F. graminearum* were also considerably high, with the diameters of inhibition zones ranging from 15.93 to 18.47 mm. The inhibition performance of 1-MT-4 was also rational, with the diameters of the inhibition zones for pathogenic bacteria and pathogenic fungi ranging from 14.83 to 19.03 mm and from 12.03 to 17.80 mm, respectively. Additionally, 1-MT-1 and 1-MT-3 exhibited poorer antimicrobial activities against pathogenic bacteria and fungi. Therefore, it can be deduced that direct addition of excessive 1-MT has negative impacts on the yield of active compounds.

**Table 3:** Antimicrobial effects of four crude extracts induced by 1-MT on pathogenic bacteria.

Crude	Inhibition zone diameter (mm)				
	*L. monocytogenes*	*E. coli*	*S. enteritidis*	*S. aureus*	*P. aeruginosa*
1-MT-1	16.93 ± 0.15	11.40 ± 0.26	13.07 ± 0.38	17.50 ± 0.87	-
1-MT-2	22.37 ± 0.45	18.27 ± 0.21	21.23 ± 0.38	22.93 ± 0.51	13.30 ± 0.20
1-MT-3	16.73 ± 0.21	10.97 ± 0.12	12.47 ± 0.35	17.43 ± 0.31	12.93 ± 0.15
1-MT-4	18.63 ± 0.15	16.20 ± 0.36	18.07 ± 0.38	19.03 ± 0.21	14.83 ± 0.31
Ampicillin sodium	33.45 ± 0.80	30.30 ± 0.72	30.97 ± 0.95	32.53 ± 0.71	23.77 ± 0.70
MeOH	-	-	-	-	-

“-” no obvious antimicrobial effect. The outer diameter of the Oxford cup was 8 mm. The concentration tested was 20 mg/mL. Three replicates were carried out for each test.

**Table 4:** Antimicrobial effects of four crude extracts induced by 1-MT on pathogenic fungi.

Crude	Inhibition zone diameter (mm)				
	*C. albicans*	*V. dahliae*	*F. moniliforme*	*F. graminearum*	*B. cinerea*
1-MT-1	11.17 ± 0.25	10.83 ± 0.06	17.30 ± 0.10	15.67 ± 1.15	15.10 ± 0.20
1-MT-2	15.93 ± 0.15	17.30 ± 1.35	21.10 ± 0.53	18.47 ± 1.31	21.83 ± 1.30
1-MT-3	10.87 ± 0.21	13.40 ± 0.66	14.57 ± 0.31	12.77 ± 1.30	13.87 ± 0.35
1-MT-4	12.03 ± 0.21	14.80 ± 0.30	17.80 ± 0.66	13.10 ± 0.56	13.83 ± 0.31
Amphotericin B	16.60 ± 0.62	20.67 ± 0.47	15.90 ± 0.80	13.97 ± 0.50	21.77 ± 0.45
MeOH	-	-	-	-	-

“-” no obvious antimicrobial effect. The outer diameter of the Oxford cup was 8 mm. The concentration tested was 20 mg/mL. Three replicates were carried out for each test.

**Figure 3:**
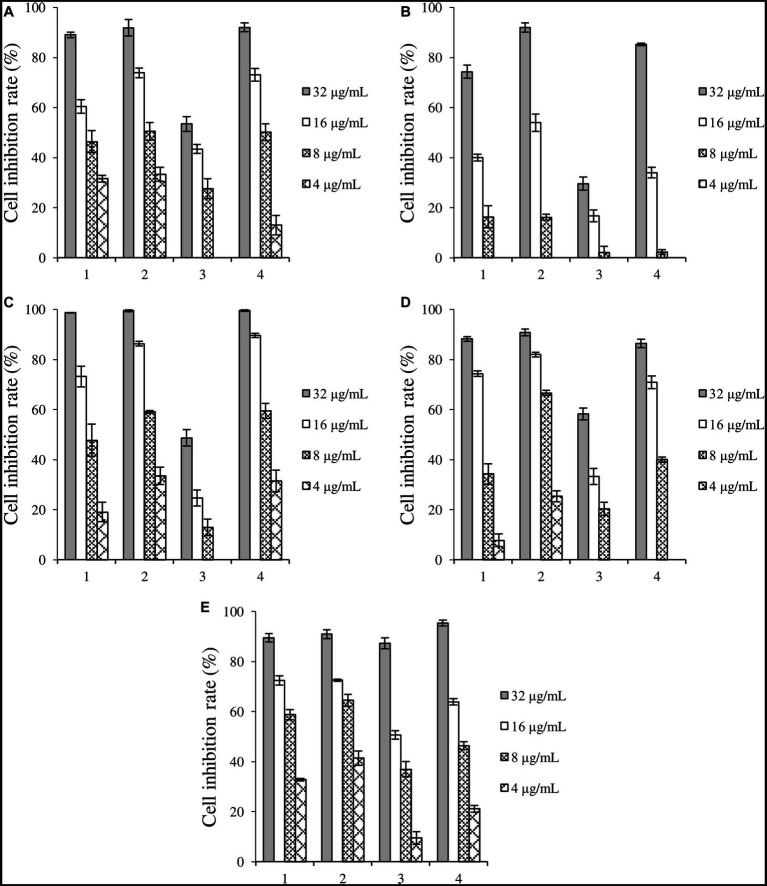
Inhibition rates of four crude extracts induced by 1-MT on tumor cells. **(A)** liver cancer cells Huh-7, **(B)** cervical cancer cell Hela, **(C)** colorectal adenocarcinoma cell HCT-15, **(D)** lung cancer cell A549, **(E)** breast cancer cells MDA-MB-231. 1: 1-MT-1, 2: 1-MT-2, 3: 1-MT-3, 4: 1-MT-4.

Crude extracts fermented under four induction conditions showed different inhibitory activities against the five cancer cells (Figure 3). 1-MT-1, 1-MT-2, and 1-MT-4 exhibited high cytotoxicity against the five cancer cells, with inhibition rates >70% at a concentration of 32 μg/mL. Among them, 1-MT-2 exhibited the highest cytotoxicity, with inhibition rates of Huh-7, HCT-15, A549 and MDA-MB-231 > 50% at a concentration of 8 μg/mL.

Overall, the optimal induction conditions were the addition of 0.25 mM 1-MT and mycelium before fermentation.

### 3.3. Compounds elucidation

As demonstrated in Figure 1, Compounds **2** (4.0 mg), **3** (3.6 mg), **4** (69.3 mg), and **5** (4.4 mg) were isolated from Fr.C; Compounds **7** (7.4 mg) and **8** (66.6 mg) were isolated from Fr.D; and Compounds **1** (62.5 mg), **6** (10.0 mg), and **9** (6.9 mg) were isolated from Fr.E.

**Figure 4:**
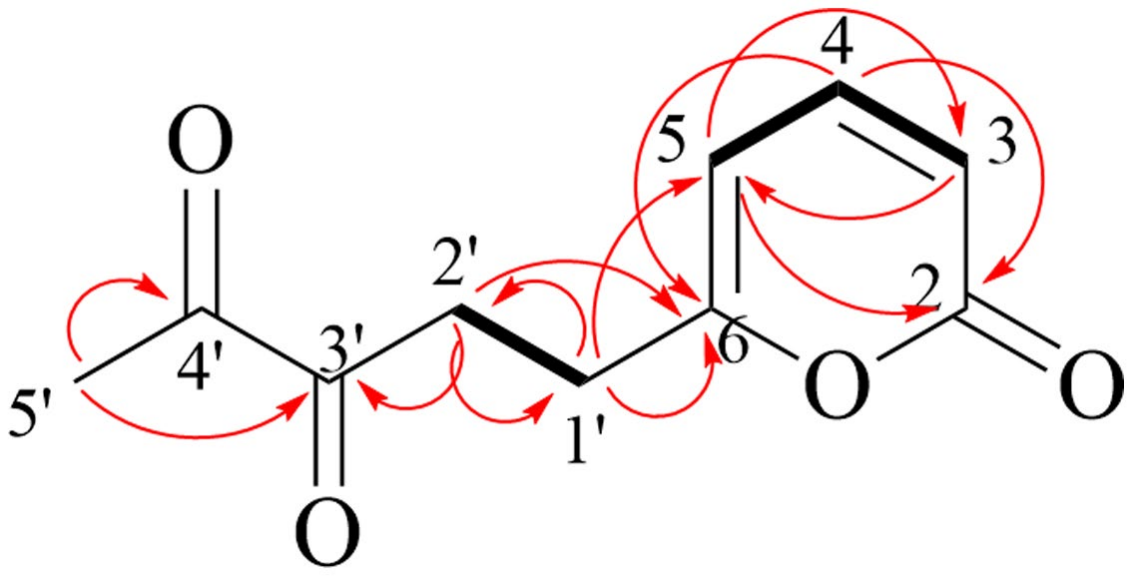
Key HMBC (red arrow) and ^1^H-^1^H COSY correlations (black bond) of compound **1**.

Compound **1** is reddish-brown oil with a molecular formula of C_10_H_10_O_4_, as deduced from HR-ESI-MS ion peak at m/z 195.0652 [M + H]^+^ (Supplementary Figure S5). The NMR spectra can be found in Supplementary Figures S6–S11. The ^13^C NMR (Table 5) and HSQC data of Compound **1** revealed the presence of 2*H*-pyran-2-one moiety, including three methine (*δ*_C_ 145.70 (4), 114.57 (3), 104.35 (5)), and two quaternary carbons (*δ*_C_166.41 (6), 162.93 (2)). The data in ^1^H NMR spectrum displayed singlets for two methylene (*δ*_H_ 3.00 (t, 7.3 Hz, 2H, 2′), 2.65 (t, 7.3 Hz, 2H, 1′)) as well as a methyl (*δ*_H_ 2.15 (s, 3H, 5′)), and that in ^13^C NMR indicated the presence of two carbonyl (*δ*_C_ 198.52 (3′), 198.14 (4′)). Key HMBC correlations (Figure 4) from H-5′ to C-4′, C-3′ established the propanedione moiety. The HMBC correlation of H-2′ with C-1′ and C-6 indicated that propanedione moiety was connected to the 2*H*-pyran-2-one moiety. Then the structure of Compound **1** was established.

**Table 5:** NMR data of compound **1** (600 MHz, Acetone-*d*_6_).

No.	**1**	
	*δ*_C_	*δ*_H_ (mult. *J*)
2	162.93	
3	114.57	5.96 (dd, 9.4, 0.9 Hz, 1H)
4	145.70	7.28 (dd, 9.4, 6.6 Hz, 1H)
5	104.35	6.06 (dq,6.6, 0.9 Hz, 1H),
6	166.41	
1’	28.60	2.65 (t, 7.3 Hz, 2H)
2’	34.03	3.00 (t, 7.3 Hz, 2H)
3’	198.52	
4’	198.14	
5’	24.31	2.15 (s, 3H)

**Figure 5:**
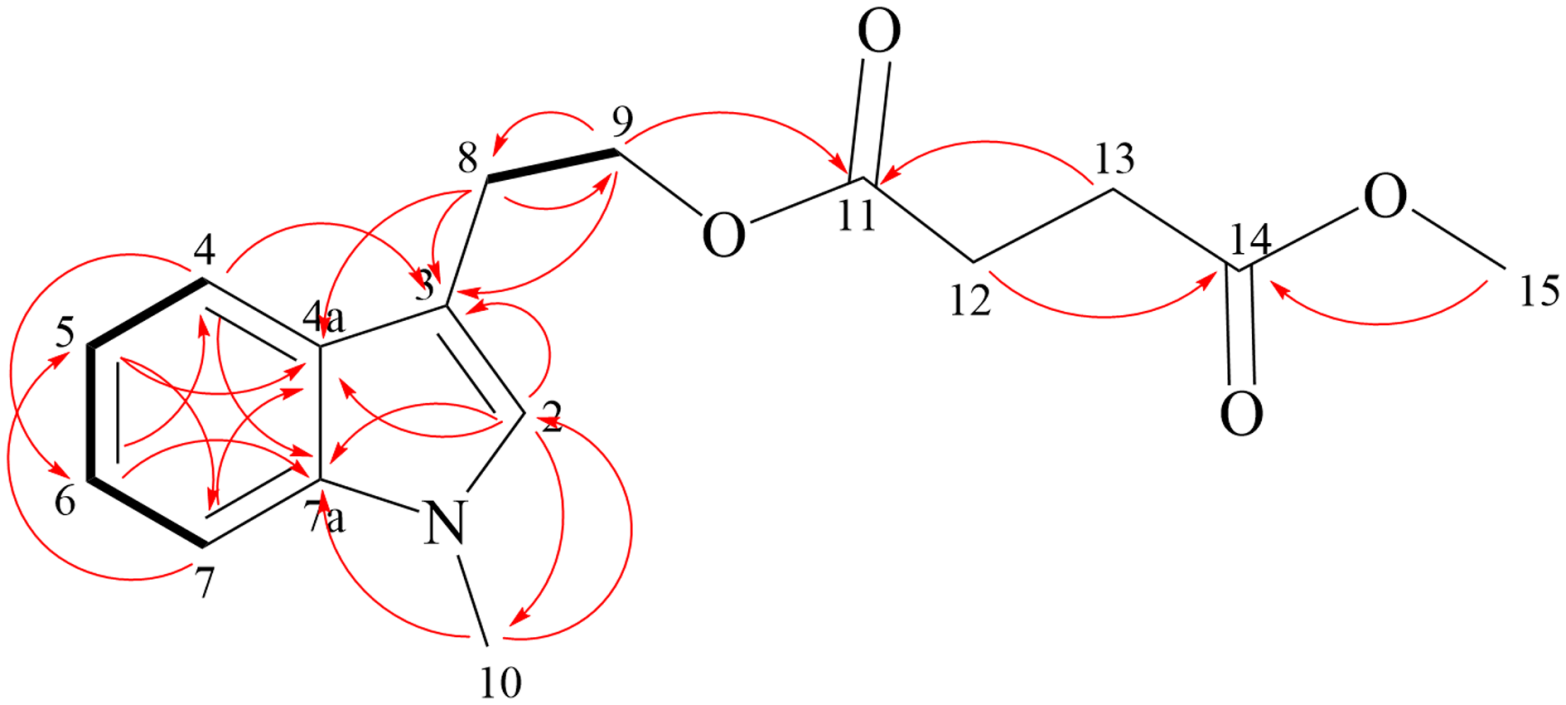
Key HMBC (red arrow) and ^1^H-^1^H COSY correlations (black bond) of compound **2**.

Compound **2** obtained as pale-yellow oil, was determined to be C_16_H_19_NO_4_ by HR-ESI-MS (m/z 312.1206 [M + Na]^+^) (Supplementary Figure S12) and was in accordance with the ^13^C NMR data (Table 6). The NMR spectra were shown in Supplementary Figures S13–S18. In the ^1^H NMR spectrum, the characteristic signals at *δ*_H_ 6.91 (s, 1H, 2), 7.61 (dt, 7.9 Hz, 1H, 4), 7.11 (ddd, 8.0, 7.0, 1.0 Hz, 1H, 5), 7.23 (ddd, 8.2, 6.9, 1.1 Hz, 1H, 6), 7.29 (d, 8.2 Hz, 1H, 7) and 3.76 (s, 3H, 10) were ascribed to a 1-methylindole moiety. The ^13^C NMR and HSQC data displayed singlets for a methyl ester (*δ*_C_ 172.33 (14), 51.85 (15)), an ethyl ester (*δ*_C_ 24.66 (8), 65.05 (9), 172.82 (11)) and two methylene (*δ*_C_ 29.20 (12), 28.92 (13)). Key HMBC correlations (Figure 5) from H-13 to C-11, and H-12 to C-14 determined the connection of two esters. The HMBC correlation of H-9 with C-3 finally established the structure of Compound **2**.

**Table 6:** NMR data of compound **2** (600 MHz, CDCl_3_).

No.	**2**	
	*δ*_C_	*δ*_H_ (mult. *J*)
2	126.90	6.91 (s, 1H)
3	110.33	
4a	127.84	
4	118.85	7.61 (dt, 7.9 Hz, 1H)
5	118.89	7.11 (ddd, 8.0, 7.0, 1.0 Hz, 1H)
6	121.65	7.23 (ddd, 8.2, 6.9, 1.1 Hz, 1H)
7	109.22	7.29 (d, 8.2 Hz, 1H),
7a	136.92	
8	24.66	3.09 (td, 7.2, 0.8 Hz, 2H),
9	65.05	4.36 (dd, 9.2, 5.2 Hz, 2H)
10	32.65	3.76 (s, 3H)
11	172.82	
12	29.20	2.61–2.66 (m, 4H)
13	28.92	
14	172.33	
15	51.85	3.68 (s, 3H)

**Figure 6:**
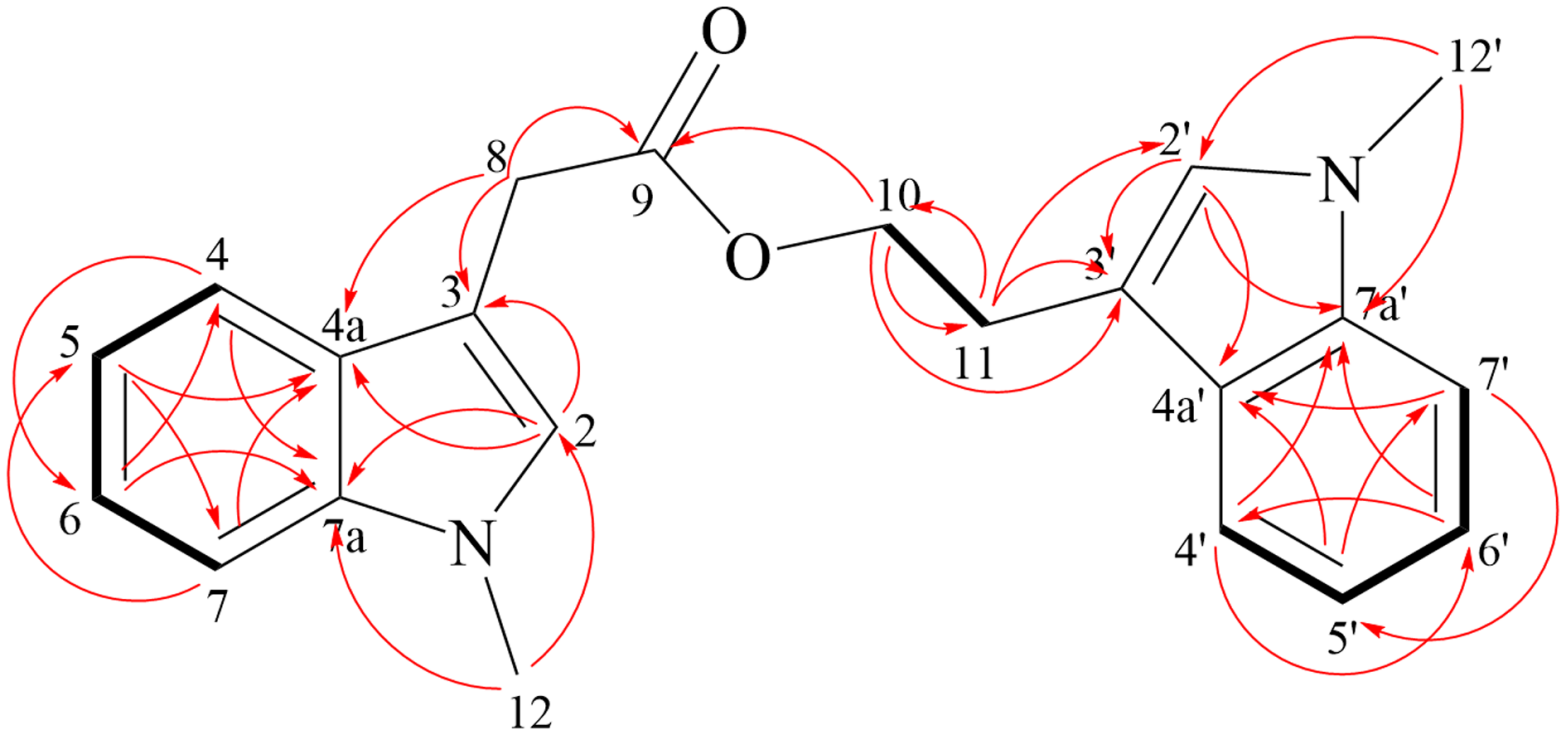
Key HMBC (red arrow) and ^1^H-^1^H COSY correlations (black bond) of compound **3**.

Compound **3** is yellow oil with a molecular formula of C_22_H_22_N_2_O_2_, as deduced from HR-ESI-MS at m/z 369.1573 [M + Na]^+^ (Supplementary Figure S19). Comparison the NMR data of Compound **3** and fusariumindole A (Guo et al., 2020; Table 7) revealed that Compound **3** had two extra methyl groups (*δ*_C_ 32.69 and *δ*_C_ 32.56). ^1^H-^1^H COSY and HMBC spectra demonstrated that the two imino groups in fusariumindole A were methylated (Supplementary Figures S20–S25). Hence, Compound **3** was identified as a methyl derivative of fusariumindole A and denoted as fusariumindole D (Figure 6).

**Table 7:** NMR data of compound **3** (600 MHz, CDCl_3_) and fusariumindole A (600 MHz, DMSO-*d*_6_).

NO.	**3**		fusariumindole A	
	*δ*_C_	*δ*_H_ (mult. *J*)	*δ*_C_	*δ*_H_ (mult. *J*)
1				10.93, (brs, 1H)
1’				10.86, (brs, 1H)
2	126.92	6.99 (s, 1H)	124.10	7.22, (d, 2.4, 1H)
2’		6.74 (s, 1H)	123.20	7.16, (d, 1.8, 1H)
3	106.88		107.00	
3’	110.45		110.00	
4a	127.76		127.10	
4a’			127.10	
4	119.13	7.60 (dd, 7.8, 1.2 Hz, 1H)	118.20	7.54, (d, 8.4 Hz, 1H)
4’	119.01	7.57 (dd, 7.8, 1.2 Hz, 1H)	118.30	7.42, (d, 8.4 Hz, 1H)
5	118.88	7.10 (t, 7.2 Hz, 1H)	118.28	6.96 (ddd, 8.4, 7.8, 0.6 Hz, 1H)
5’	118.83	7.10 (t, 7.2 Hz, 1H)	118.28	6.98 (ddd, 8.4, 7.8, 0.6 Hz, 1H)
6	121.71	7.23 (t, 7.8 Hz, 1H)	121.00	7.07 (ddd, 8.4, 7.8, 1.2 Hz, 1H)
6’	121.56	7.22 (t, 7.8 Hz, 1H)	120.95	7.07 (ddd, 8.4, 7.8, 1.2 Hz, 1H)
7	109.22	7.30 (d, 8.4 Hz, 1H)	111.30	7.35, (d, 8.4 Hz, 1H)
7’	109.16	7.28 (d, 8.4 Hz, 1H)	111.30	7.35, (d, 8.4 Hz, 1H)
7a	136.86		136.10	
7a’			136.20	
8	31.35	3.77 (s, 2H)	31.30	3.73, (s, 2H)
9	172.18		171.60	
10	65.08	4.37 (t, 7.2 Hz, 2H)	64.40	4.27 (t, 7.2 Hz, 2H)
11	24.64	3.08 (t, 7.2 Hz, 2H)	24.10	3.00 (t, 7.2 Hz, 2H)
12	32.69	3.74 (s, 3H)		
12’	32.56	3.67 (s, 3H)		

**Figure 7:**
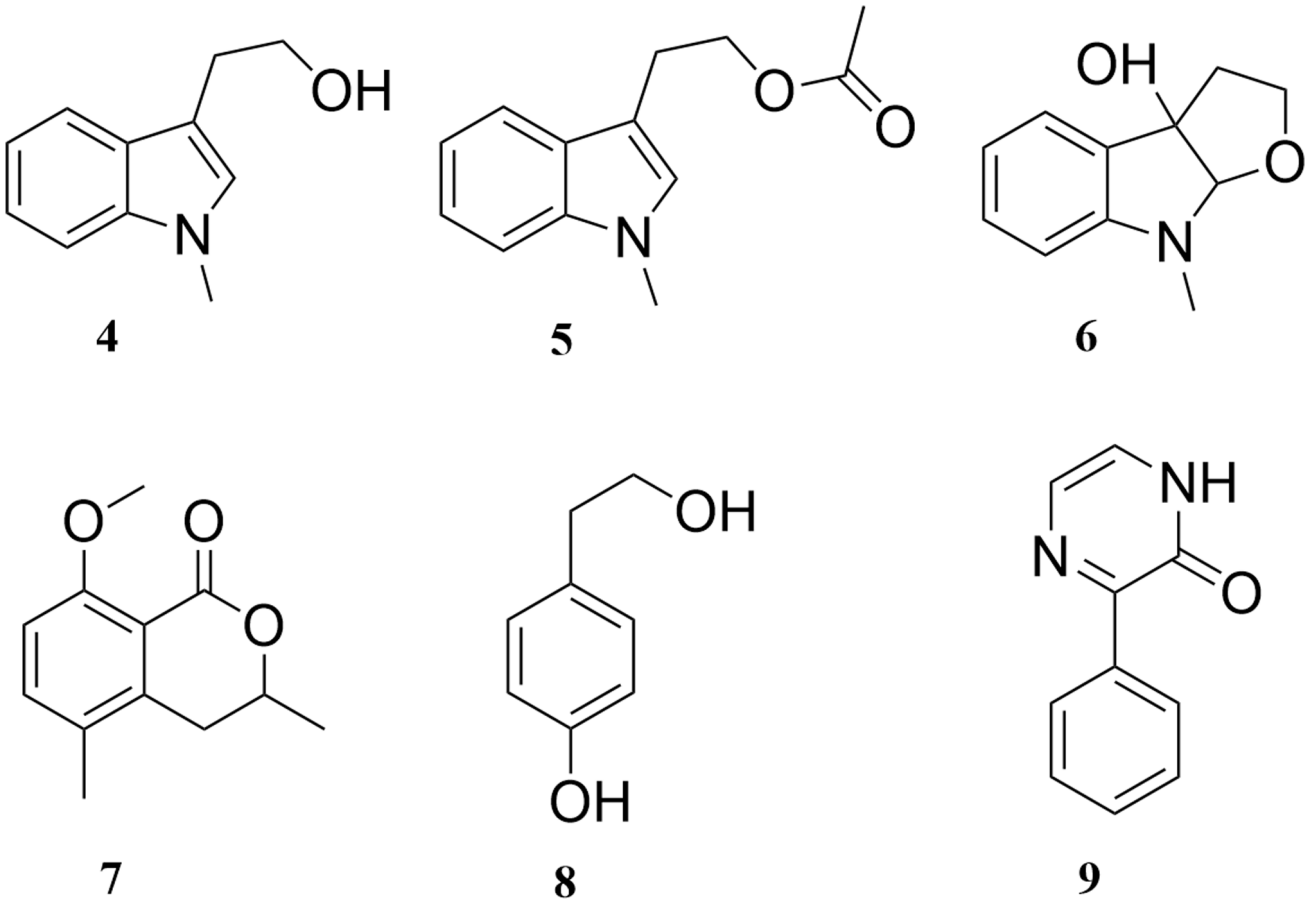
Structures of compounds **4**–**9.**

Based on the NMR data of compounds **4–9** in the Supplementary Figures S26–S37, six known compounds were identified as *N*-methyl tryptophol (**4**) (Liu et al., 2012), 3-(2-acetoxyethyl)-1-methylindole (**5**) (Leeson, 1984; Shaker et al., 2021), *N*-methyl-3a-hydroxyfuroindoline (**6**) (Hirose et al., 2005), 3,5-dimethyl-8-methoxy-3,4-dihydroisocoumarin (**7**) (Kamisuki et al., 2007), p-hydroxyphenethyl alcohol (**8**) (Jang et al., 2014), 3-phe-nylpyrazin-2(1*H*)-one (**9**) (El Euch et al., 2018). The structures of compounds **4–9** are shown in Figure 7.

Conventional culture of *Diaporthe* sp. AC1 led to identification of eight compounds with no indole alkaloids (Gu et al., 2022), while in this study, five derivatives of the 1-MT (Compounds **2**–**6**) were obtained. Researches have demonstrated that 1-MT not only could elicit the production of Pictet-Spengler reaction-based alkaloids (Yan et al., 2014), but can also be converted into novel indoles through biotransformation by *Chaetomium globosum* (Yan et al., 2019). The results herein proved that 1-MT could also be transformed by *Diaporthe* sp. AC1. The indole structure motif occurs in a variety of natural products as well as in synthetic drugs (Hu et al., 2021). This nucleus is associated with a broad spectrum of biological profiles ranging from antifungal and antibacterial to cytotoxic activities (Zhang et al., 2014; Li et al., 2016; Han et al., 2020). Hence, the compounds were used to determine antimicrobial and cytotoxic activities.

### 3.4. Antimicrobial activity of compounds

**Figure 8:**
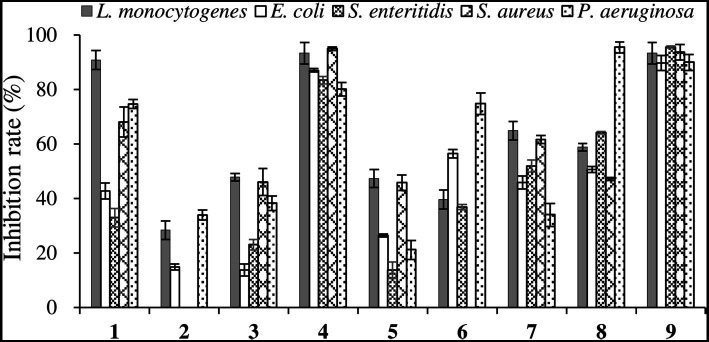
Inhibition rates of six compounds on pathogenic bacteria.

The antibacterial activities of the compounds against pathogenic bacteria were relatively weak, thus only the antibacterial effect at the concentration of 512 μg/mL was shown. As displayed in Figure 8, among the new compounds, Compound **1** exhibited high inhibition rate (> 90%) against *L. monocytogenes*, while Compounds **2** and **3** exhibited low inhibitory activities against pathogenic bacteria. For the known compounds, Compounds **4** and **9** exhibited good antibacterial activities against five pathogenic bacteria with all inhibition rates above 80%, and Compound **8** showed promising inhibitory performance on *P. aeruginosa*, with an inhibition rate > 90%. As reported before, Compound **9** showed good inhibitory activity against *S. aureus*, *L. monocytogenes*, and *S. typhimurium* (El Euch et al., 2018). The results of this study demonstrated that Compound **9** also had significant antibacterial activity against *S. enterica*, *P. aeruginosa*, and *E. coli*, indicating that this compound has broad-spectrum antibacterial activity.

The MIC values of the compounds against the five pathogenic fungi was shown in Table 8. Compound **1** showed different inhibitory activities against four pathogenic fungi, with MIC values ranging from 128 to 512 μg/mL. Compounds **2** and **3** showed inhibitory activity against only one pathogenic fungus. Compound **4** exhibited inhibitory activity against five pathogenic fungi, with MIC against *F. moniliforme*, *F. graminearum*, and *B. cinerea* being 128 μg/mL.

**Table 8:** Antifungal activities of compounds **1**–**9** and amphotericin B.

Compounds	Pathogenic fungi (MIC μg/mL)				
	*C. albicans*	*V. dahliae*	*F. moniliforme*	*F. graminearum*	*B. cinerea*
**1**	-	256	512	128	256
**2**	-	-	-	-	256
**3**	-	512	-	-	-
**4**	256	512	128	128	128
**5**	-	512	-	-	256
**6**	-	-	-	128	512
**7**	-	512	-	256	-
**8**	-	256	-	256	512
**9**	-	-	512	256	512
Amphotericin B	32	16	32	16	16

“-” MIC > 512 μg/mL. Three replicates were carried out for each test.

Overall, Compound **4** exhibited inhibition activities against both pathogenic bacteria and pathogenic fungi. However, Compounds **2**, **3**, **5** and **6**, which are derivatives of Compound **4**, showed low antibacterial activity, indicating that 11-OH is a key functional group. The bioactivity of *N*-methyl tryptophol (**4**) was few reported, but the antifungal and antibacterial activities of tryptophol have been proved (Li et al., 1994; Jin et al., 2015; Singkum et al., 2019), indicating the negligible effect of N-methyl on the activity.

### 3.5. Cytotoxicity of compounds

As shown in Table 9, the inhibitory effects of different compounds on cancer cells were significantly different. Compound **1** showed high cytotoxicity against all cancer cells. The IC_50_ of (S,E)-6-(4-hydroxy-3-oxopent-1-en-1-yl)-2*H*-pyran-2-one (an analog of Compound **1**) against A549 and MDA-MB-231 was 84.5 and 64.3 μM, respectively (Gu et al., 2022), while in this study, the IC_50_ of Compound **1** against the two cancer cells was 28.20 and 12.26 μM, respectively. Structurally, the hydroxyl group in the C-4′ position of (S,E)-6-(4-hydroxy-3-oxopent-1-en-1-yl)-2H-pyran-2-one changed into a carbonyl group, demonstrating that the carbonyl group of C-4′ in compound **1** was extremely important for the enhanced cytotoxicity.

**Table 9:** Cytotoxicity of compounds **1**–**9** and doxorubicin hydrochloride (DOX).

Compounds	IC_50_ (μM)				
	Huh-7	Hela	HCT-15	A549	MDA-MB-231
**1**	29.70 ± 3.35	52.52 ± 3.62	13.60 ± 1.43	28.20 ± 1.77	12.26 ± 1.43
**2**	-	-	-	-	170.68 ± 12.81
**3**	-	130.66 ± 12.48	184.05 ± 9.50	184.69 ± 17.47	80.46 ± 1.56
**4**	133.74 ± 13.67	29.74 ± 1.30	101.88 ± 8.50	95.80 ± 2.50	71.63 ± 1.88
**5**	180.06 ± 1.34	137.76 ± 12.55	-	133.48 ± 14.32	144.82 ± 10.23
**6**	-	139.43 ± 7.09	-	-	163.12 ± 3.69
**7**	-	118.23 ± 9.81	-	106.66 ± 3.38	129.52 ± 6.12
**8**	-	120.42 ± 16.56	-		-
**9**	168.00 ± 1.44	-	-	135.88 ± 1.79	159.13 ± 9.60
DOX	0.22 ± 0.02	0.18 ± 0.05	0.17 ± 0.01	0.23 ± 0.01	0.27 ± 0.02

“-” IC_50_ > 200 μM.

Compound **4** showed moderate cytotoxicity against Hela (IC_50_ = 29.74 μM). Among the derivatives of 1-MT (Compounds **2–6**), only Compound **4** showed relatively good inhibitory activity against the cancer cells, further demonstrating the key role of the hydroxyl group at C11 in Compound **4**.

## 4. Conclusion

By feeding 1-MT into cultures of the endophytic fungus *Diaporthe* sp. AC1 to increase the diversity of metabolites, three novel compounds (**1**–**3**), together with six known ones were isolated and characterized. Most of the compounds were the biotransformation products of 1-MT. In the bioactivity assay, Compound **1** showed moderate antibacterial activity against *V. dahlia*, *F. graminearum,* and *B. cinerea*, with MIC values being 256, 128, and 256 μg/mL, respectively. It also showed high cytotoxicity against five cancer cells, with IC_50_ ranging from 12.26 to 52.52 μM. Other compounds also exhibited certain antimicrobial and cytotoxic activities. Under the severe situation that antibiotic resistance is a growing public threat, this work provides an efficient and eco-friendly methodology for the development of structurally undescribe compounds, from which lead molecules can be recognized and optimized for new drugs discovery.

## Data availability statement

The original contributions presented in the study are included in the article/Supplementary material, further inquiries can be directed to the corresponding authors.

## Author contributions

SZ: Writing – original draft, Investigation. QX: Writing – original draft, Investigation. CJ: Writing – original draft, Investigation. XH: Investigation, Writing – original draft. YZ: Investigation, Writing – original draft. CL: Investigation, Writing – original draft. LM: Investigation, Writing – original draft. WS: Investigation, Writing – original draft. YL: Writing – original draft, Formal analysis. ZY: Formal analysis, Writing – review & editing. FZ: Writing – review & editing, Project administration. YT: Project administration, Writing – original draft.

## References

[ref1] Chen C. J. Li D. Yu K. Y. Zhou M. M. (2021). Generation of tryptamine derivatives through biotransformation by *Diaporthe* sp. J. Asian Nat. Prod. Res. 23, 1164–1170. doi: 10.1080/10286020.2020.1866560 33432842

[ref2] Chepkirui C. Stadler M. (2017). The genus *Diaporthe*: a rich source of diverse and bioactive metabolites. Mycol. Prog. 16, 477–494. doi: 10.1007/s11557-017-1288-y

[ref3] Deshmukh S. K. Gupta M. K. Prakash V. Saxena S. (2018). Endophytic fungi: a source of potential antifungal compounds. J. Fungi. 4:77. doi: 10.3390/jof4030077, PMID: 29941838 PMC6162562

[ref4] El Euch I. Z. Frese M. Sewald N. Smaoui S. Shaaban M. Mellouli L. (2018). Bioactive secondary metabolites from new terrestrial *streptomyces* sp. TN82 strain: isolation, structure elucidation and biological activity. Med. Chem. Res. 27, 1085–1092. doi: 10.1007/s00044-017-2130-4

[ref5] Gakuubi M. M. Ching K. C. Munusamy M. Wibowo M. Liang Z. X. Kanagasundaram Y.. (2022). Enhancing the discovery of bioactive secondary metabolites from fungal endophytes using chemical elicitation and variation of fermentation media. Front. Microbiol. 13:898976. doi: 10.3389/fmicb.2022.898976 35733953 PMC9207341

[ref6] Gao H. Li G. Lou H. X. (2018). Structural diversity and biological activities of novel secondary metabolites from endophytes. Molecules 23:646. doi: 10.3390/molecules23030646, PMID: 29534010 PMC6017594

[ref7] Gokhale M. Gupta D. Gupta U. Faraz R. Sandhu S. S. (2017). Patents on endophytic fungi. Recent Pat. Biotechnol. 11, 120–140. doi: 10.2174/1872208311666170215151834 28215141

[ref8] Gu H. Zhang S. Liu L. Yang Z. Zhao F. Tian Y. (2022). Antimicrobial potential of endophytic fungi from *Artemisia argyi* and bioactive metabolites from *Diaporthe* sp. AC1. Front. Microbiol. 13:908836. doi: 10.3389/fmicb.2022.908836, PMID: 35814687 PMC9260665

[ref9] Guo Y. W. Liu X. J. Yuan J. Li H. J. Mahmud T. Hong M. J.. (2020). L-tryptophan induces a marine-derived *fusarium* sp. to produce indole alkaloids with activity against the zika virus. J. Nat. Prod. 83, 3372–3380. doi: 10.1021/acs.jnatprod.0c00717, PMID: 33180497

[ref10] Han Y. Dong W. Guo Q. Li X. Huang L. (2020). The importance of indole and azaindole scaffold in the development of antitumor agents. Eur. J. Med. Chem. 203:112506. doi: 10.1016/j.ejmech.2020.112506, PMID: 32688198

[ref11] Hautbergue T. Jamin E. L. Debrauwer L. Puel O. Oswald I. P. (2018). From genomics to metabolomics, moving toward an integrated strategy for the discovery of fungal secondary metabolites. Nat. Prod. Rep. 35, 147–173. doi: 10.1039/C7NP00032D, PMID: 29384544

[ref12] Hirose T. Sunazuka T. Yamamoto D. Kojima N. Shirahata T. Harigaya Y.. (2005). Determination of the absolute stereochemistry and asymmetric total synthesis of madindolines a and B: a practical improvement to a second – generation approach from the first – generation. Tetrahedron 61, 6015–6039. doi: 10.1016/j.tet.2005.04.056

[ref13] Hu Y. Chen S. Yang F. Dong S. (2021). Marine indole alkaloids—isolation, structure and bioactivities. Mar. Drugs 19:658. doi: 10.3390/md19120658 34940657 PMC8708922

[ref14] Jang M. S. Park H. Y. Nam K. H. (2014). Whitening effects of 4-hydroxyphenethyl alcohol isolated from water boiled with *Hizikia fusiformis*. Food Sci. Biotechnol. 23, 555–560. doi: 10.1007/s10068-014-0076-6

[ref15] Jin M. Xu C. Zhang X. (2015). The effect of tryptophol on the bacteriophage infection in high-temperature environment. Appl. Microbiol. Biot. 99, 8101–8111. doi: 10.1007/s00253-015-6674-2, PMID: 25994257

[ref16] Kamisuki S. Ishimaru C. Onoda K. Kuriyama I. Ida N. Sugawara F.. (2007). Nodulisporol and Nodulisporone, novel specific inhibitors of human DNA polymerase λ from a fungus, *Nodulisporium* sp. Bioorgan. Med. Chem. 15, 3109–3114. doi: 10.1016/j.bmc.2007.02.052, PMID: 17363259

[ref17] Leeson P. D. (1984). A synthetic route to dehydrosecodine analogues. J. Chem. Soc. Perkin Trans. 1, 2125–2128. doi: 10.1039/P19840002125

[ref18] Li H. Y. Matsunaga S. Fusetani N. (1994). Simple antifungal metabolites from a marine sponge, *Halichondria* sp. Comp. Biochem. Phys. Part B 107, 261–264. doi: 10.1016/0305-0491(94)90048-5

[ref19] Li C. Shao Y. Li W. Yin T. Li H. Yan H.. (2022). Hybrid diterpenic meroterpenoids from an endophytic *penicillium* sp. induced by chemical epigenetic manipulation. J. Nat. Prod. 85, 1486–1494. doi: 10.1021/acs.jnatprod.1c01155 35658485

[ref20] Li M. C. Sun W. S. Cheng W. Liu D. Liang H. Zhang Q. Y.. (2016). Four new minor brominated indole related alkaloids with antibacterial activities from *Laurencia similis*. Bioorg. Med. Chem. Lett. 26, 3590–3593. doi: 10.1016/j.bmcl.2016.06.015, PMID: 27318539

[ref21] Liu M. Grkovic T. Liu X. Han J. Zhang L. Quinn J. R. (2017). A systems approach using OSMAC, log P and NMR fingerprinting: an approach to novelty. Syn. Syst. Biotechno. 2, 276–286. doi: 10.1016/j.synbio.2017.10.001, PMID: 29552652 PMC5851912

[ref22] Liu C. Zhang W. Dai L. X. You S. L. (2012). Cascade dearomatization of N-substituted tryptophols via Lewis acid-catalyzed Michael reactions. Org. Biomol. Chem. 10, 7177–7183. doi: 10.1039/C2OB26139A 22850826

[ref23] Mosmann T. (1983). Rapid colorimetric assay for cellular growth and survival: application to proliferation and cytotoxicity assays. J. Immunol. Methods 65, 55–63. doi: 10.1016/0022-1759(83)90303-4, PMID: 6606682

[ref24] Pinedo-Rivilla C. Aleu J. Durán-Patrón R. (2022). Cryptic metabolites from marine – derived microorganisms using OSMAC and epigenetic approaches. Mar. Drugs 20:84. doi: 10.3390/md20020084, PMID: 35200614 PMC8879561

[ref25] Ranuka T. H. Thammarat A. Chulabhorn M. Somsak R. Prasat K. (2014). One strain – many compounds (OSMAC) method for production of polyketides, azaphilones, and an isochromanone using the endophytic fungus *Dothideomycete* sp. Phytochemistry 108, 87–94. doi: 10.1016/j.phytochem.2014.09.013 25310919

[ref26] Shaker S. Fan R. Z. Li H. J. Lan W. J. (2021). A pair of novel bisindole alkaloid enantiomers from marine fungus *fusarium* sp. XBB-9. Nat. Prod. Res. 35, 1497–1503. doi: 10.1080/14786419.2019.1655416, PMID: 31437009

[ref27] Singkum P. Muangkaew W. Suwanmanee S. Pumeesat P. Wongsuk T. Luplertlop N. (2019). Suppression of the pathogenicity of *Candida albicans* by the quorum-sensing molecules farnesol and tryptophol. J. Gen. Appl. Microbio. 65, 277–283. doi: 10.2323/jgam.2018.12.002, PMID: 31217414

[ref28] Siridechakorn I. Yue Z. Mittraphab Y. Lei X. Pudhom K. (2017). Identification of spirobisnaphthalene derivatives with anti – tumor activities from the endophytic fungus *Rhytidhysteron rufulum* AS21B. Bioorgan. Med. Chem. 25, 2878–2882. doi: 10.1016/j.bmc.2017.02.054, PMID: 28274675

[ref29] Uzma F. Mohan C. D. Hashem A. Konappa N. M. Rangappa S. Kamath P. V.. (2018). Endophytic fungi – alternative sources of cytotoxic compounds: a review. Front. Pharmacol. 9:309. doi: 10.3389/fphar.2018.00309, PMID: 29755344 PMC5932204

[ref30] Wang G. Ran H. Fan J. Keller N. P. Liu Z. Wu F.. (2022). Fungal – fungal cocultivation leads to widespread secondary metabolite alteration requiring the partial loss – of – function VeA1 protein. Sci. Adv. 8:eabo6094. doi: 10.1126/sciadv.abo6094, PMID: 35476435 PMC9045611

[ref31] White J. F. Kingsley K. L. Zhang Q. Verma R. Obi N. Dvinskikh S.. (2019). Review: endophytic microbes and their potential applications in crop management. Pest Manag. Sci. 75, 2558–2565. doi: 10.1002/ps.5527, PMID: 31228333 PMC6771842

[ref32] Yan W. Ge H. M. Wang G. Jiang N. Mei Y. N. Jiang R.. (2014). Pictet – Spengler reaction – based biosynthetic machinery in fungi. Proc. Natl. Acad. Sci. U. S. A. 111, 18138–18143. doi: 10.1073/pnas.1417304111, PMID: 25425666 PMC4280624

[ref33] Yan W. Zhao S. S. Ye Y. H. Zhang Y. Y. Zhang Y. Xu J. Y.. (2019). Generation of indoles with agrochemical significance through biotransformation by *Chaetomium globosum*. J. Nat. Prod. 82, 2132–2137. doi: 10.1021/acs.jnatprod.8b01101 31329433

[ref34] Zgoda J. R. Porter J. R. (2001). A convenient microdilution method for screening natural products against bacteria and fungi. Pharm. Biol. 39, 221–225. doi: 10.1076/phbi.39.3.221.5934

[ref35] Zhang L. Hua Z. Song Y. Feng C. (2014). Monoterpenoid indole alkaloids from *Alstonia rupestris* with cytotoxic, antibacterial and antifungal activities. Fitoterapia 97, 142–147. doi: 10.1016/j.fitote.2014.05.018, PMID: 24887700

