# Supplementary material for: Study on secondary metabolites of endophytic fungus Diaporthe sp. AC1 induced by tryptophan analogs
Source: Front Microbiol. 2023 Oct 9;14:1254609. doi: 10.3389/fmicb.2023.1254609 (PMC10591187; doi:10.3389/fmicb.2023.1254609)
Supplement: Supplementary file 1 [file Data_Sheet_1.pdf]

Supplementary Material:

Study on secondary metabolites of endophytic fungus *Diaporthe* sp. AC1 induced by tryptophan analogs

Shikai Zhang[1,†], Qing Xu[1,†], Changbo Ji[2,†], Xiaoyu Han[2], Yang Zhou[2], Chao Liang[2], Linran Ma[2], Weijian Sun[2], Yanling Li[2], Zhengyou Yang[1], Fengchun Zhao[1,*], Yuan Tian[2,*]

1. Department of Microbiology, College of Life Science, Key Laboratory for Agriculture Microbiology, Shandong Agricultural University, Taian 271018, China

2. College of Life Science, Shandong First Medical University & Shandong Academy of Medical Sciences, Taian 271016, China

[*] Corresponding Author
Fengchun Zhao zhaofengchun@sdau.edu.cn
Yuan Tian ytian@sdfmu.edu.cn
†These authors have contributed equally to this work.

Table S1 Yields and characteristics of four kinds of crude extracts.
Table S2 Yields and characteristics of four crude extracts induced by 1-MT.
Figure S1 Antimicrobial effects of four kinds of crude extracts on pathogenic bacteria.
Figure S2 Antimicrobial effects of four kinds of crude extracts on pathogenic fungi.
Figure S3 Antimicrobial effects of four crude extracts induced by 1-MT on pathogenic bacteria.
Figure S4 Antimicrobial effects of four crude extracts induced by 1-MT on pathogenic fungi.
Figure S5 The HR-MS of compound **1**.
Figure S6 The ^{1}H NMR spectrum of compound **1** (Acetone-d_6, 600 MHz).
Figure S7 The ^{13}C NMR spectrum of compound **1** (Acetone-d_6, 151 MHz).
Figure S8 The ^{1}H-^{1}H COSY spectrum of compound **1** (Acetone-d_6, 600 MHz).
Figure S9 The HSQC spectrum of compound **1** (Acetone-d_6, 600 MHz).
Figure S10 The HMBC spectrum of compound **1** (Acetone-d_6, 600 MHz).
Figure S11 The NOESY spectrum of compound **1** (Acetone-d_6, 600 MHz).
Figure S12 The HR-MS of compound **2**.
Figure S13 The ^{1}H NMR spectrum of compound **2** ($CDCl_3$, 600 MHz).
Figure S14 The ^{13}C NMR spectrum of compound **2** ($CDCl_3$, 151 MHz).
Figure S15 The ^{1}H-^{1}H COSY spectrum of compound **2** ($CDCl_3$, 600 MHz).
Figure S16 The HSQC spectrum of compound **2** ($CDCl_3$, 600 MHz).
Figure S17 The HMBC spectrum of compound **2** ($CDCl_3$, 600 MHz).
Figure S18 The NOESY spectrum of compound **2** ($CDCl_3$, 600 MHz).
Figure S19 The HR-MS of compound **3**.
Figure S20 The ^{1}H NMR spectrum of compound **3** ($CDCl_3$, 600 MHz).
Figure S21 The ^{13}C NMR spectrum of compound **3** ($CDCl_3$, 151 MHz).
Figure S22 The ^{1}H-^{1}H COSY spectrum of compound **3** ($CDCl_3$, 600 MHz).
Figure S23 The HSQC spectrum of compound **3** ($CDCl_3$, 600 MHz).
Figure S24 The HMBC spectrum of compound **3** ($CDCl_3$, 600 MHz).
Figure S25 The NOESY spectrum of compound **3** ($CDCl_3$, 600 MHz).
Figure S26 The ^{1}H NMR spectrum of compound **4** (Acetone-d_6, 600 MHz).
Figure S27 The ^{13}C NMR spectrum of compound **4** (Acetone-d_6, 151 MHz).
Figure S28 The ^{1}H NMR spectrum of compound **5** (Acetone-d_6, 600 MHz).
Figure S29 The ^{13}C NMR spectrum of compound **5** (Acetone-d_6, 151 MHz).
Figure S30 The ^{1}H NMR spectrum of compound **6** ($CDCl_3$, 600 MHz).
Figure S31 The ^{13}C NMR spectrum of compound **6** ($CDCl_3$, 151 MHz).
Figure S32 The ^{1}H NMR spectrum of compound **7** (Acetone-d_6, 600 MHz).
Figure S33 The ^{13}C NMR spectrum of compound **7** (Acetone-d_6, 151 MHz).
Figure S34 The ^{1}H NMR spectrum of compound **8** (Acetone-d_6, 600 MHz).
Figure S35 The ^{13}C NMR spectrum of compound **8** (Acetone-d_6, 151 MHz).
Figure S36 The ^{1}H NMR spectrum of compound **9** ($CDCl_3$, 600 MHz).
Figure S37 The ^{13}C NMR spectrum of compound **9** ($CDCl_3$, 151 MHz).

Table S1 Yields and characteristics of four kinds of crude extracts.

Number	Yield (mg/L)	Colour	State
CK	150.0	Reddish brown	Lump
5-HTP	296.5	Black	Paste
1-MT	253.0	Brown	Paste
TA	305.0	Black	Paste

CK: crude extract without tryptophan analogs, 5-HTP: crude extract added with 5-HTP, 1-MT:crude extract added with 1-MT, TA: crude extract added with TA.

Table S2 Yields and characteristics of four crude extracts induced by 1-MT.

Number	Yield (mg/L)	Colour	State
1-MT-1	242.0	Black	Paste
1-MT-2	251.0	Brown	Paste
1-MT-3	240.0	Black	Paste
1-MT-4	253.0	Brown	Paste

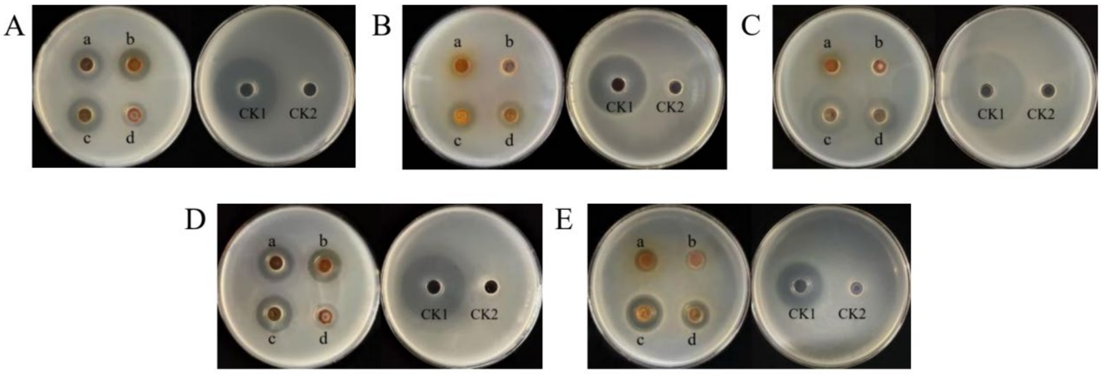

Figure S1 Antimicrobial effects of four kinds of crude extracts on pathogenic bacteria.

A: *L. monocytogenes*, **B**: *E. coli*, **C**: *S. enteritidis*, **D**: *S. aureus*, **E**: *P. aeruginosa.* a: crude extract without tryptophan analogs, b: crude extract added with 5-HTP, c: crude extract added with 1-MT, d: crude extract added with TA, CK1: ampicillin sodium, CK2: MeOH.

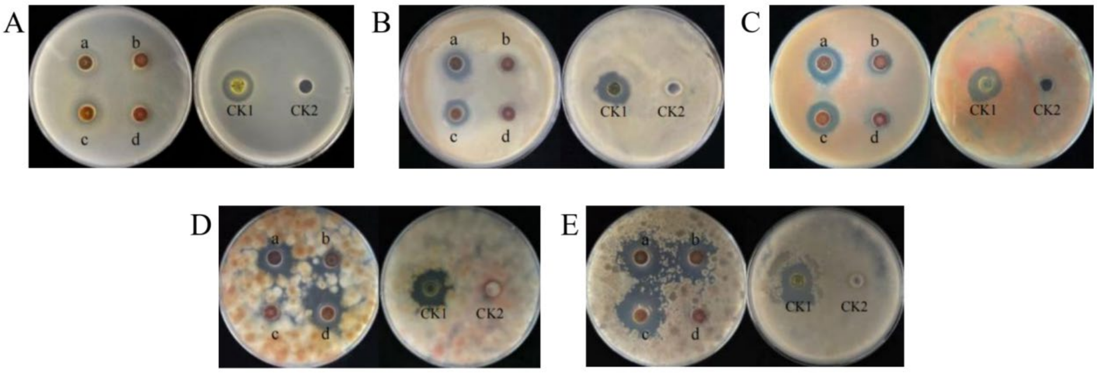

Figure S2 Antimicrobial effects of four kinds of crude extracts on pathogenic fungi.

A: *C. albicans*, **B**: *V. dahlia*, **C**: *F. moniliforme*, **D**: *F. graminearum*, **E**: *B. cinerea*, a: crude extract without tryptophan analogs, b: crude extract added with 5-HTP, c: crude extract added with 1-MT, d: crude extract added with TA, CK1: amphotericin B, CK2: MeOH.

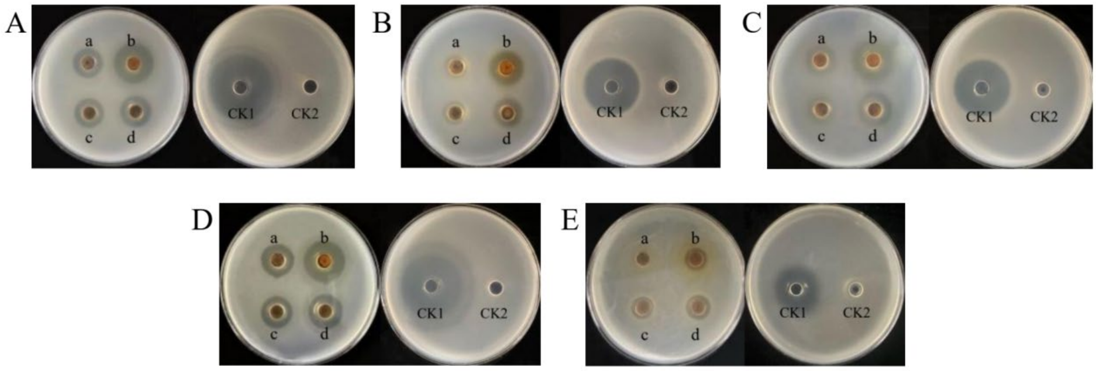

Figure S3 Antimicrobial effects of four crude extracts induced by 1-MT on pathogenic bacteria.

A: *L. monocytogenes*, **B**: *E. coli*, **C**: *S. enteritidis*, **D**: *S. aureus*, **E**: *P. aeruginosa*, a: 1-MT-1, b: 1-MT-2, c: 1-MT-3, d: 1-MT-4, CK1: ampicillin sodium, CK2: MeOH.

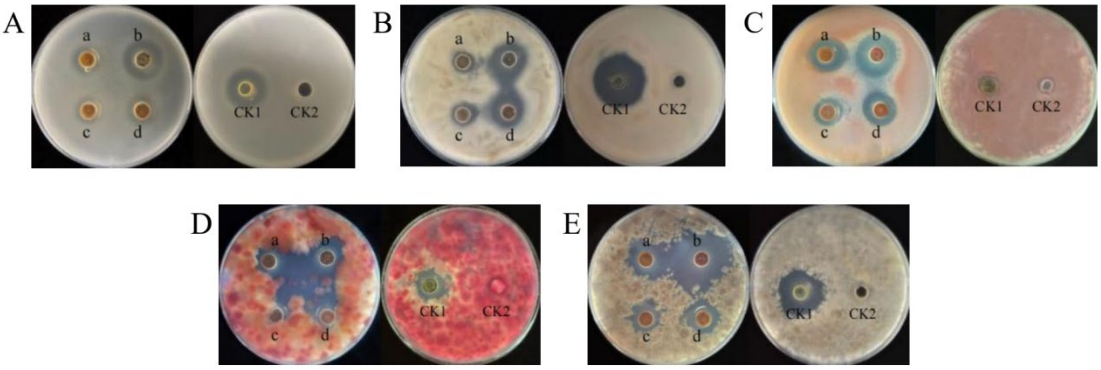

Figure S4 Antimicrobial effects of four crude extracts induced by 1-MT on pathogenic fungi.

A: *C. albicans*, B: *V. dahlia*, C: *F. moniliforme*, D: *F. graminearum*, **E**: *B. cinerea*, a: 1-MT-1, b: 1-MT-2, c: 1-MT-3, d: 1-MT-4, CK1: amphotericin B, CK2: MeOH.

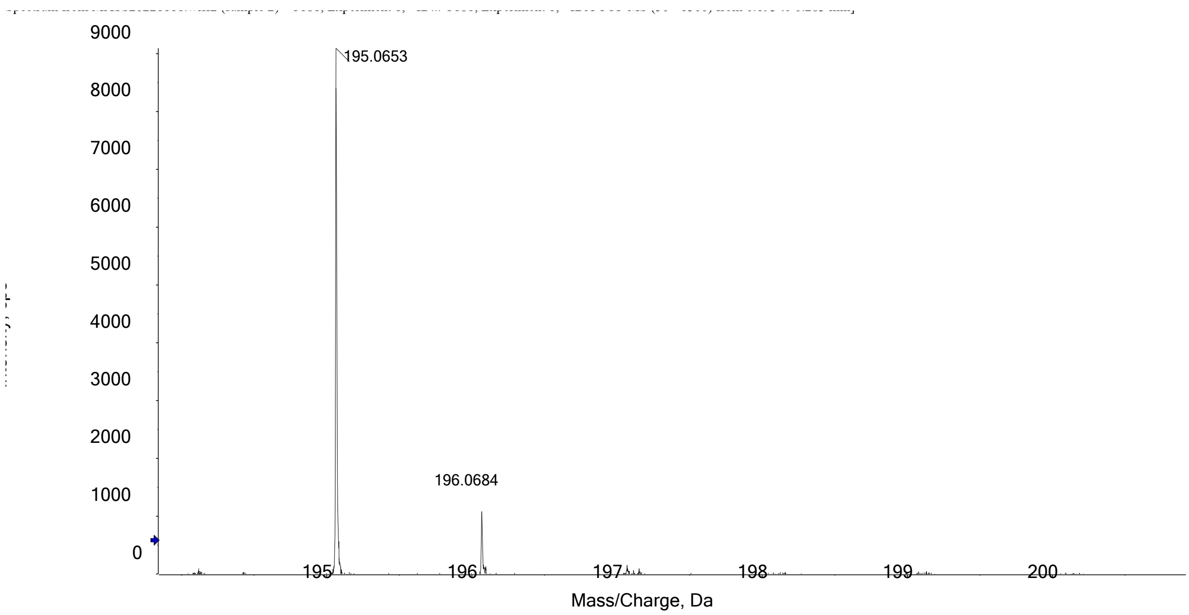

Figure S5 The HR-MS of compound **1**.

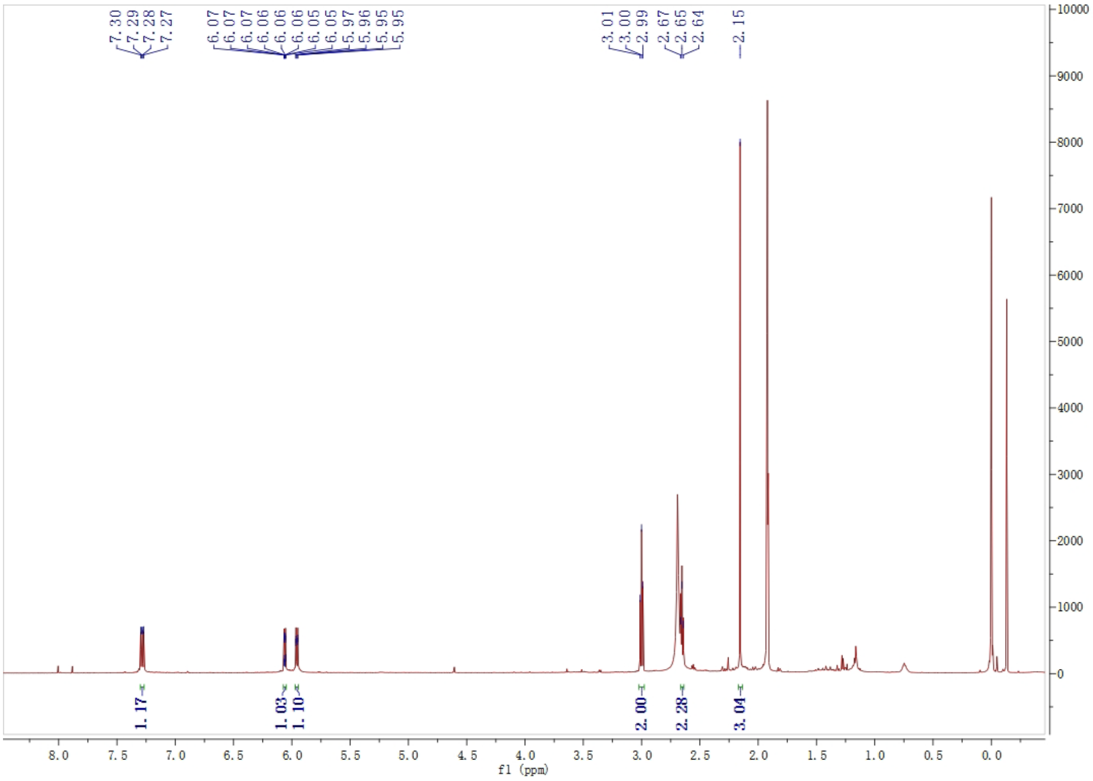

Figure S6 The ^{1}H NMR spectrum of compound **1** (Acetone-d_6, 600 MHz).

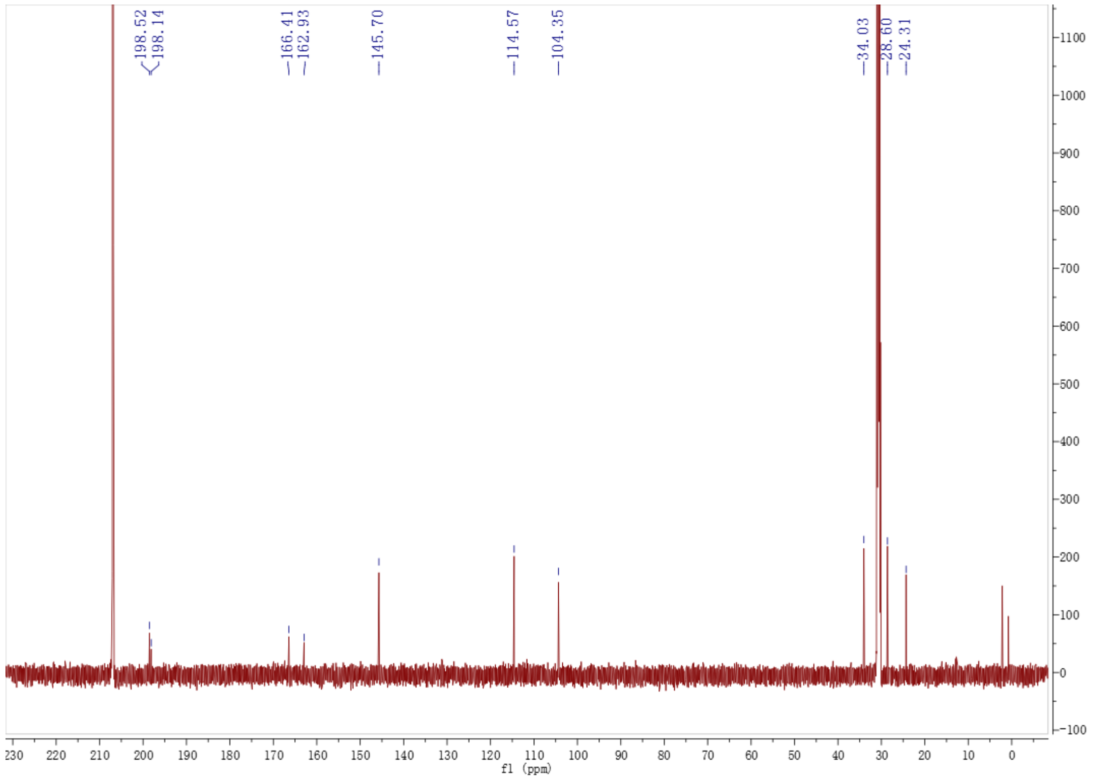

Figure S7 The ^{13}C NMR spectrum of compound **1** (Acetone-d_6, 151 MHz).

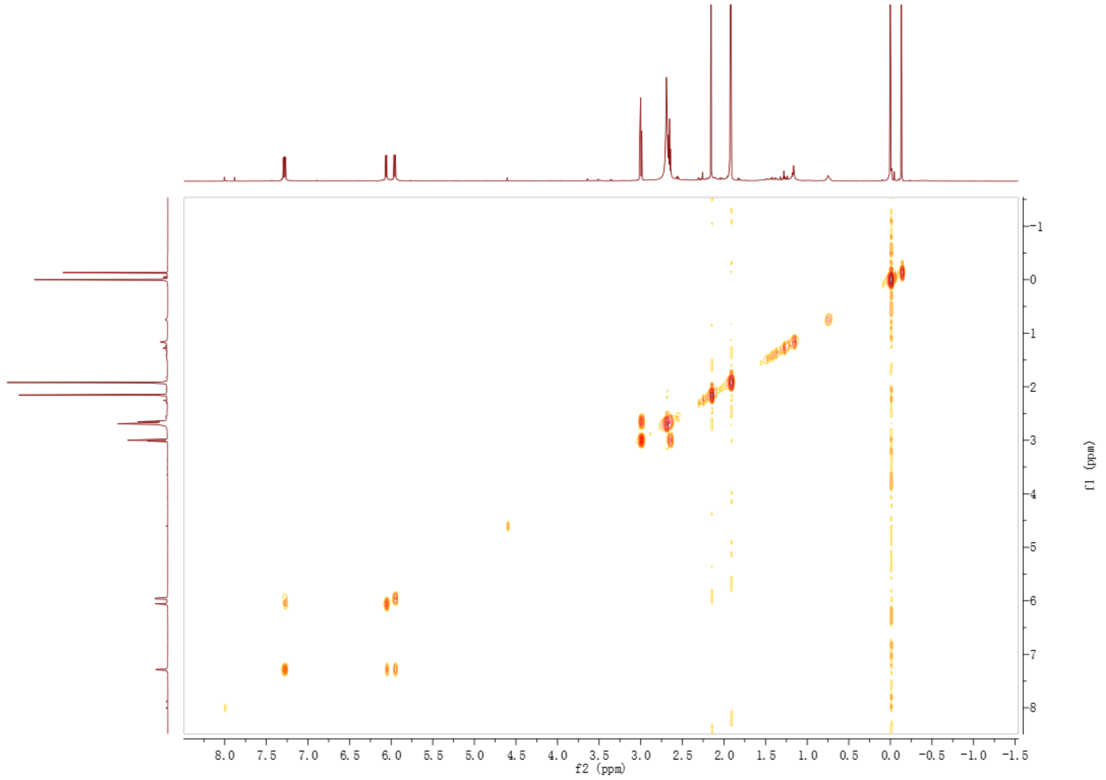

Figure S8 The ^{1}H-^{1}H COSY spectrum of compound **1** (Acetone-d_6, 600 MHz).

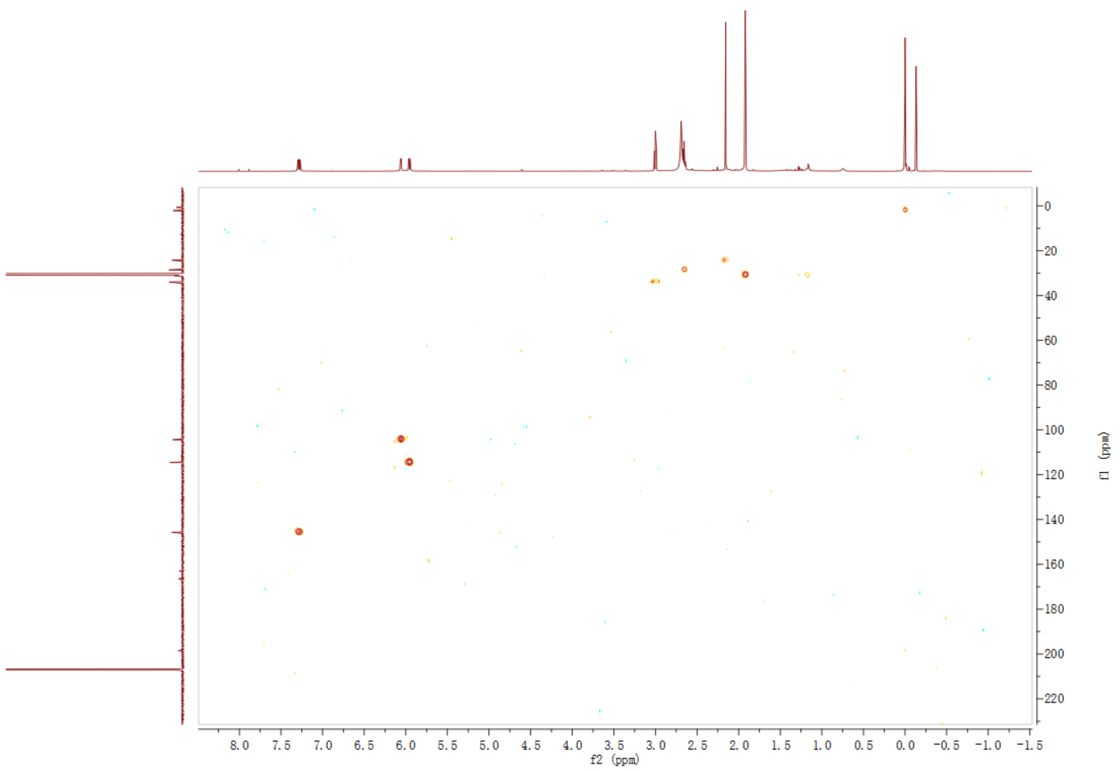

Figure S9 The HSQC spectrum of compound **1** (Acetone-d_6, 600 MHz).

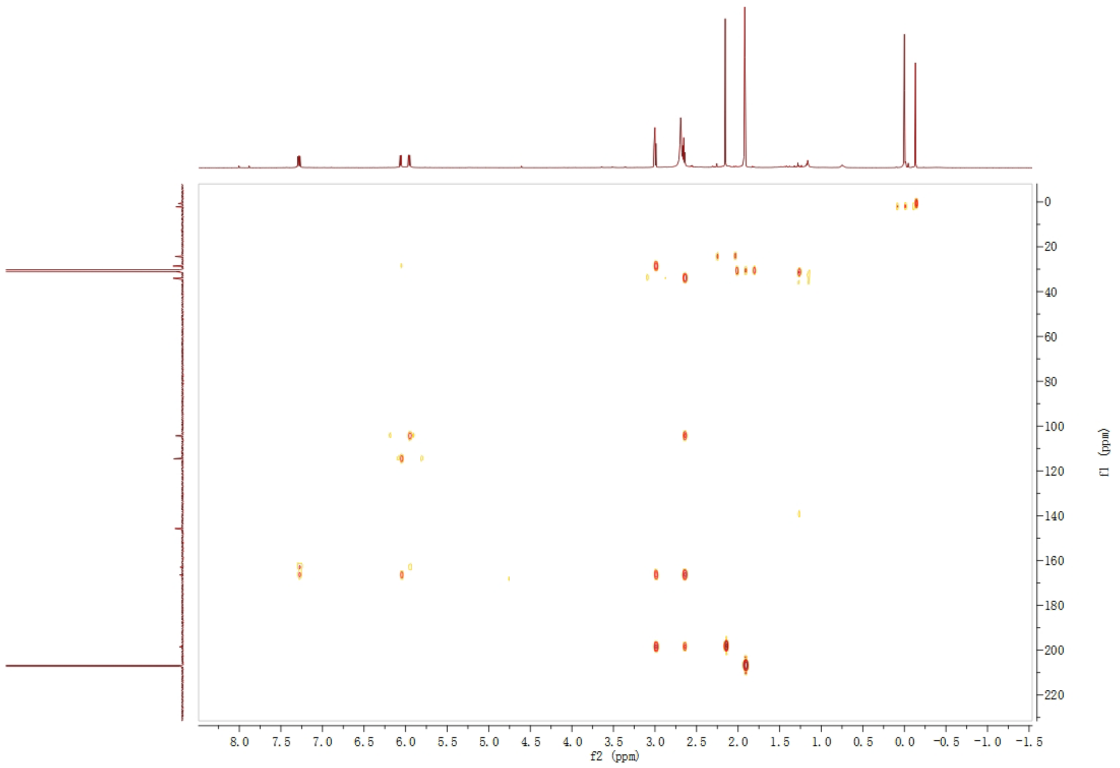

Figure S10 The HMBC spectrum of compound **1** (Acetone-d_6, 600 MHz).

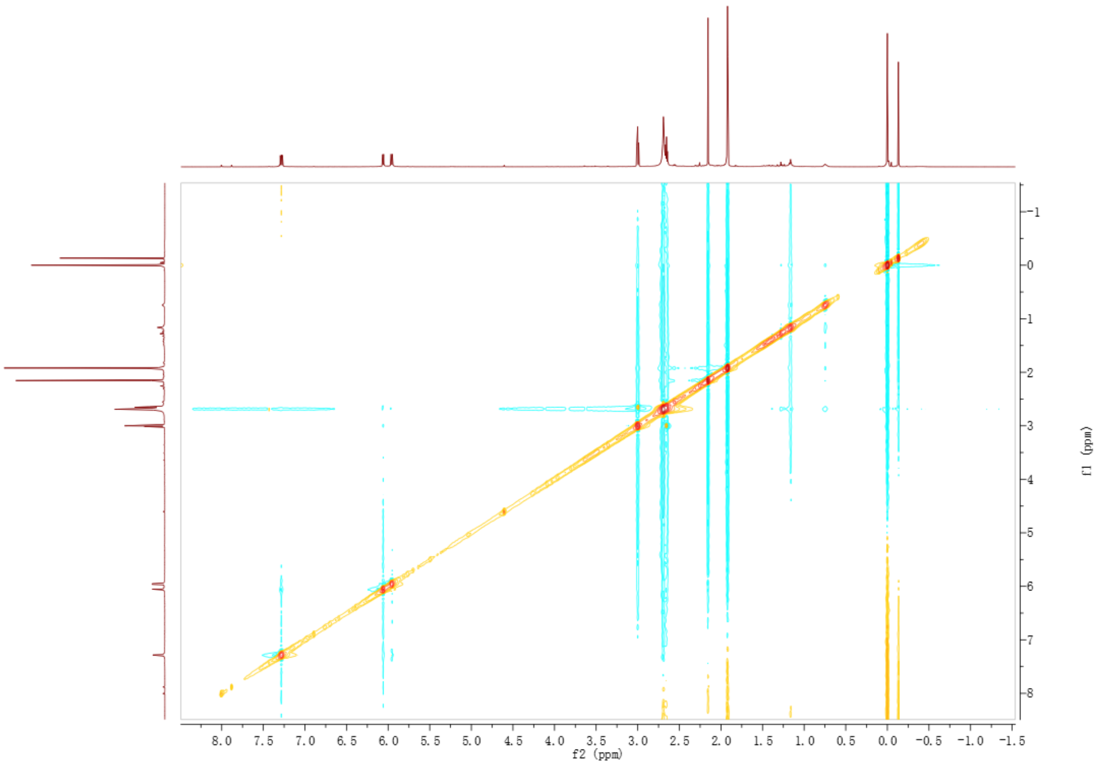

Figure S11 The NOESY spectrum of compound **1** (Acetone-d_6, 600 MHz).

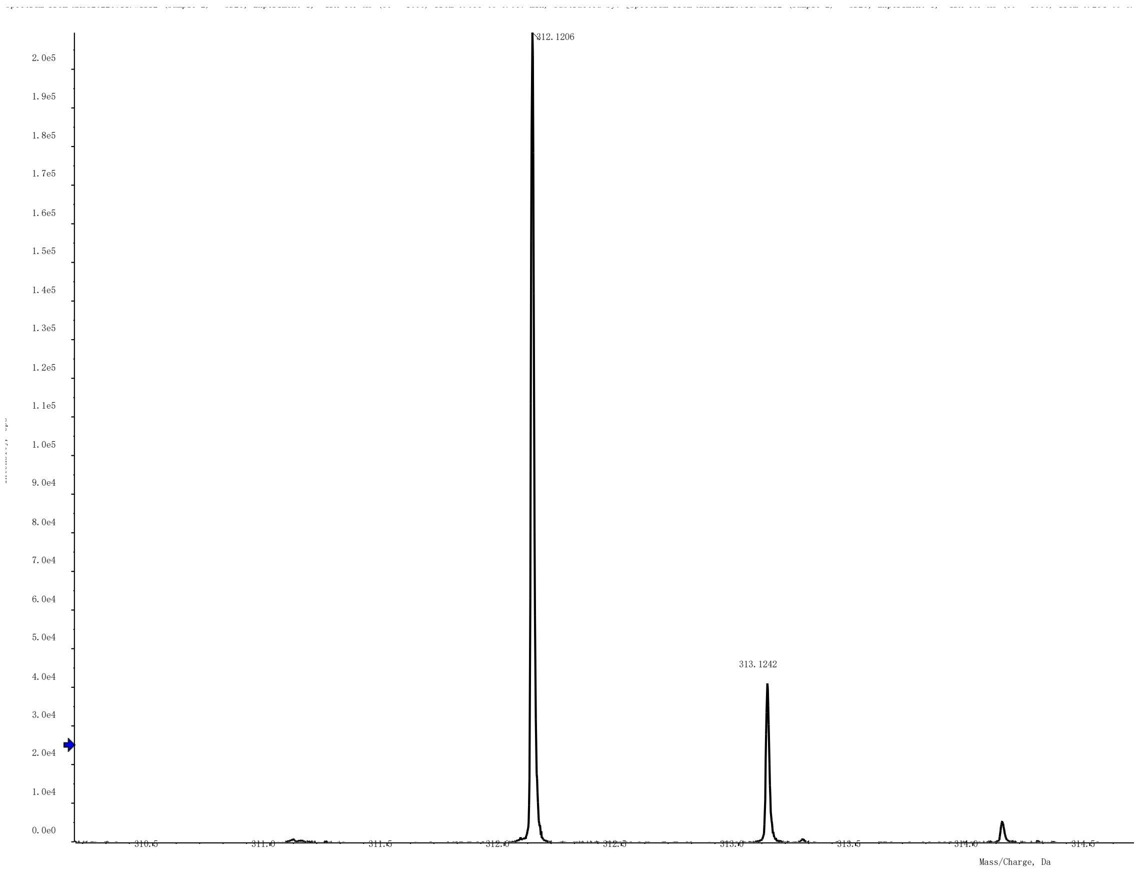

Figure S12 The HR-MS of compound **2**.

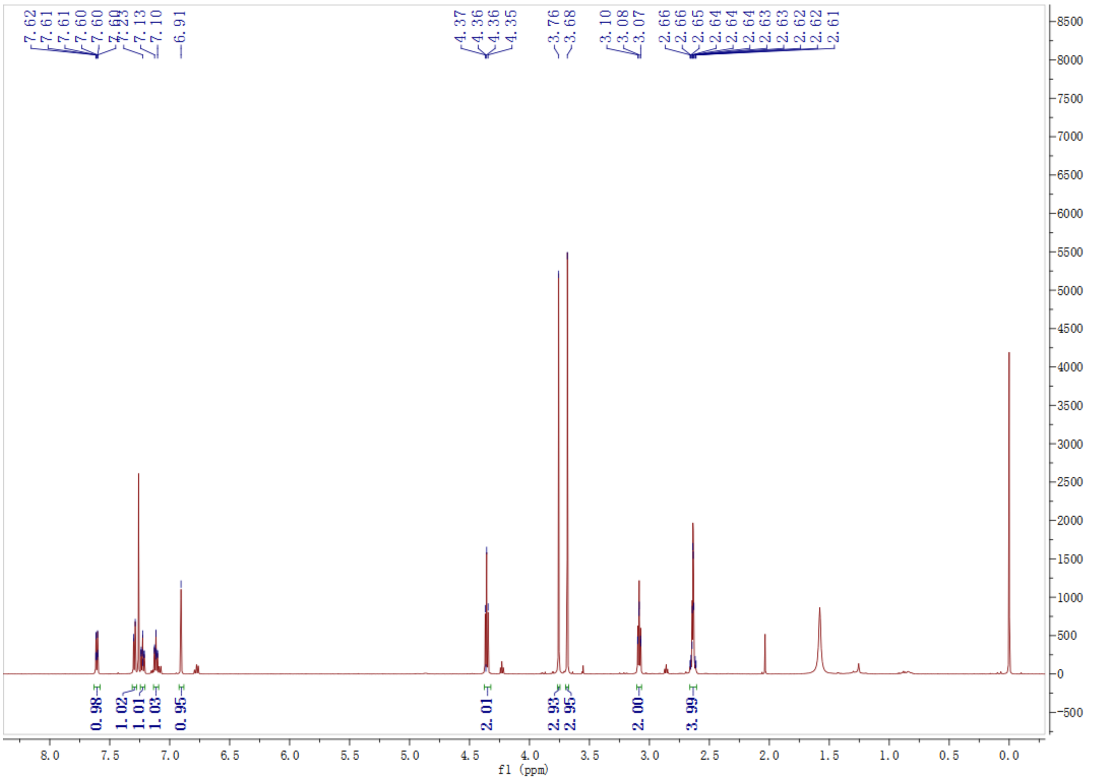

Figure S13 The ^{1}H NMR spectrum of compound **2** ($CDCl_3$, 600 MHz).

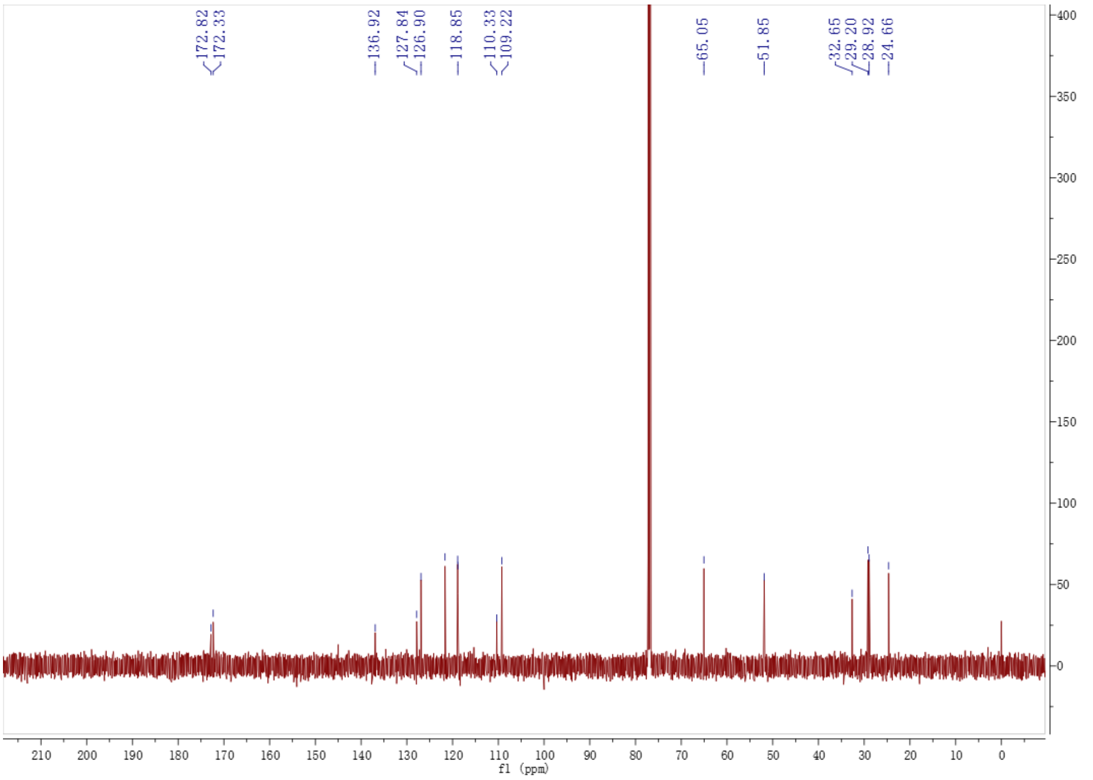

Figure S14 The ^{13}C NMR spectrum of compound **2** ($CDCl_3$, 151 MHz).

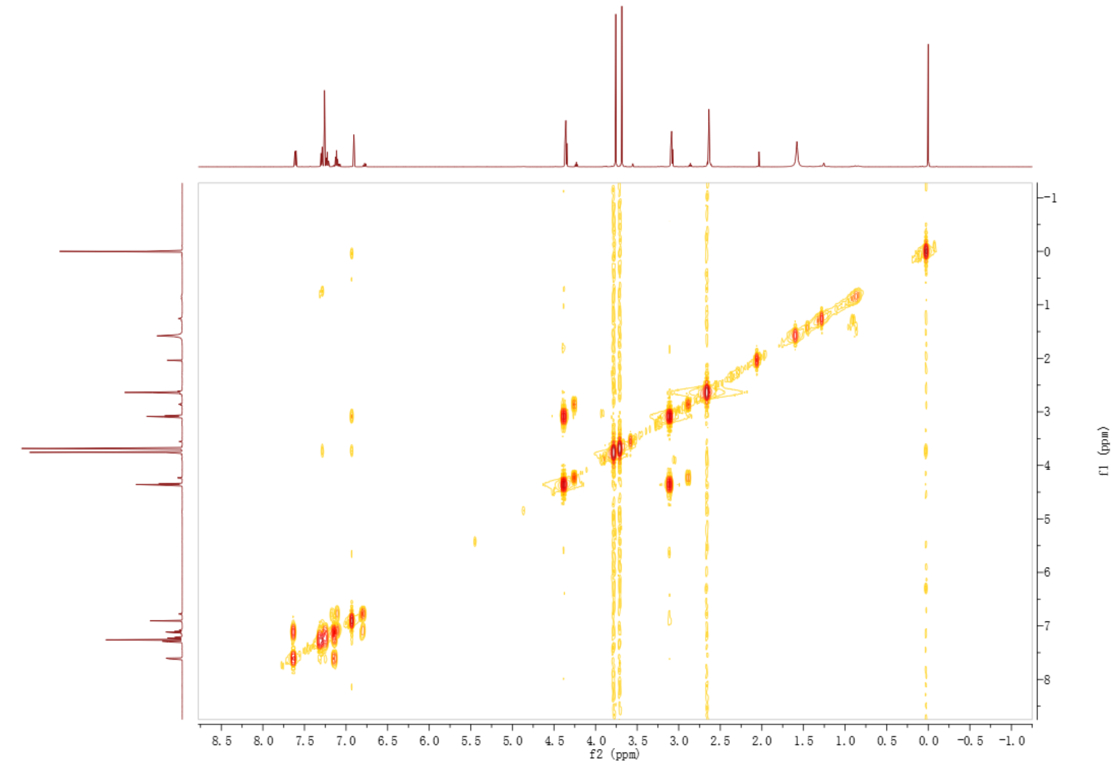

Figure S15 The ^1H-^1H COSY spectrum of compound **2** ($CDCl_3$, 600 MHz).

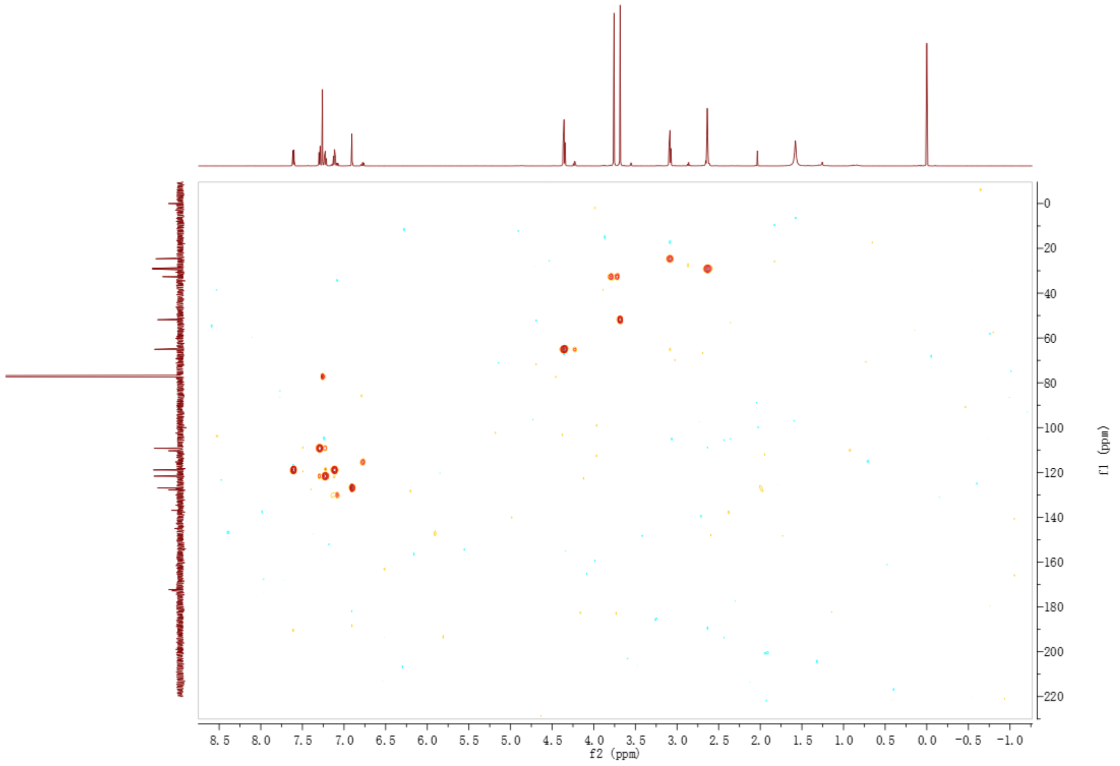

Figure S16 The HSQC spectrum of compound **2** ($CDCl_3$, 600 MHz).

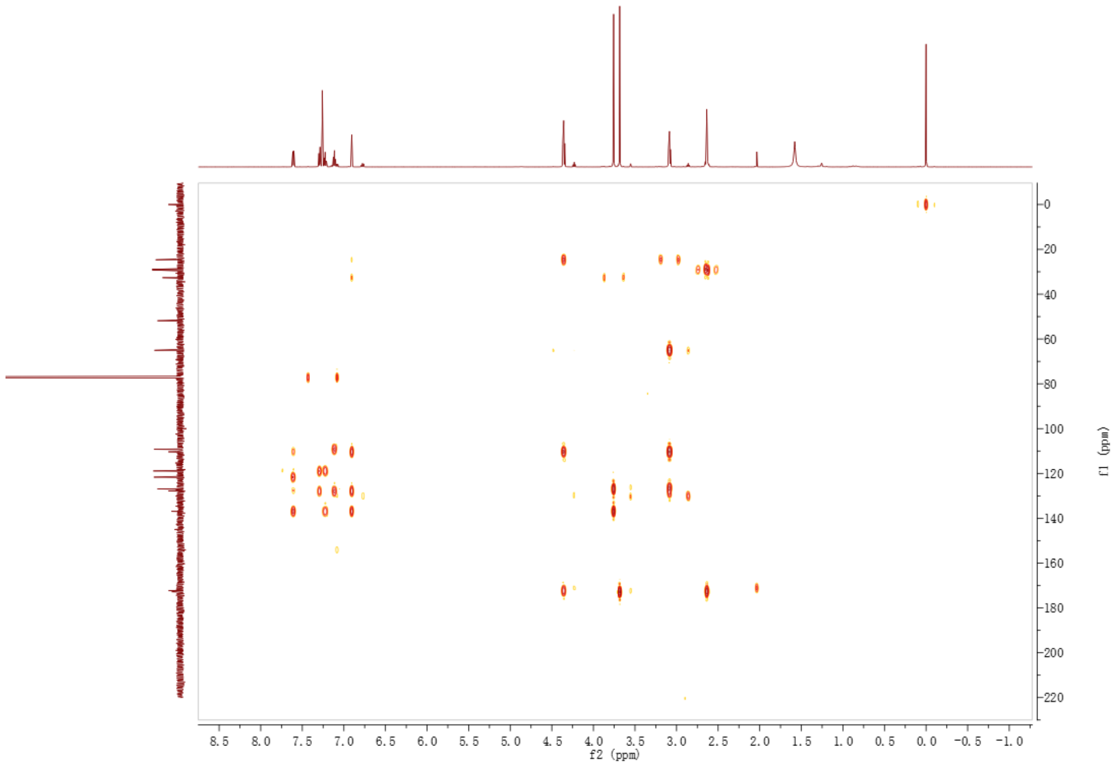

Figure S17 The HMBC spectrum of compound **2** ($CDCl_3$, 600 MHz).

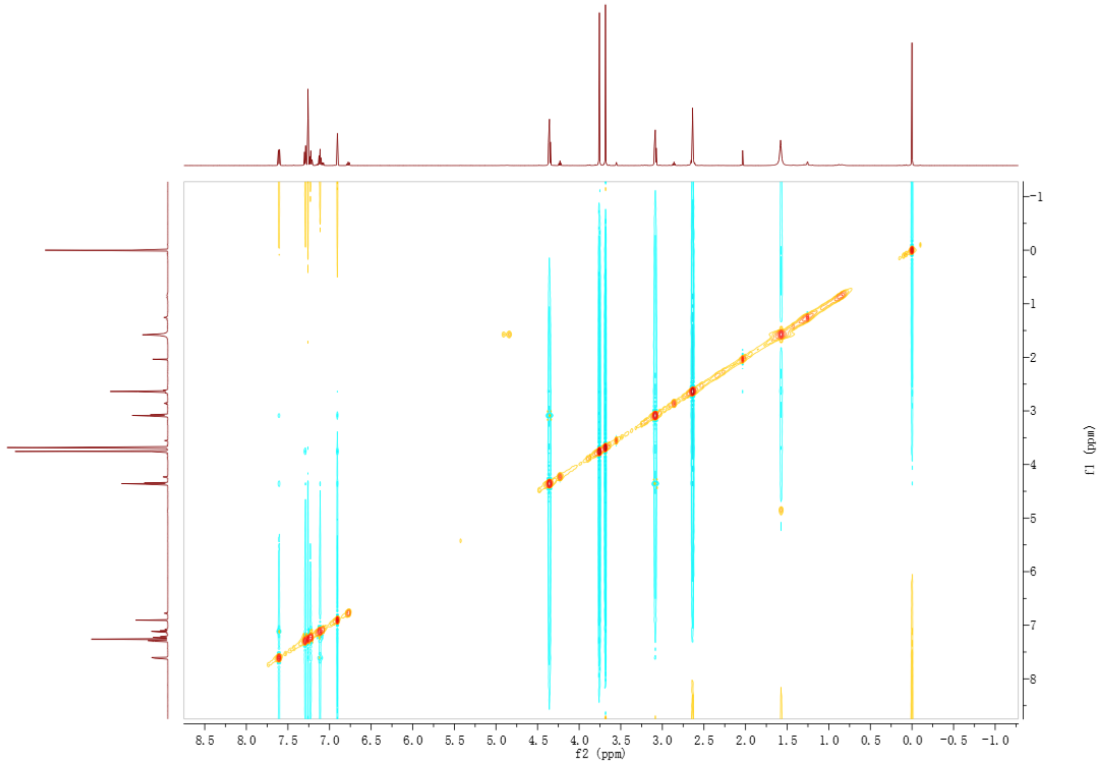

Figure S18 The NOESY spectrum of compound **2** ($CDCl_3$, 600 MHz).

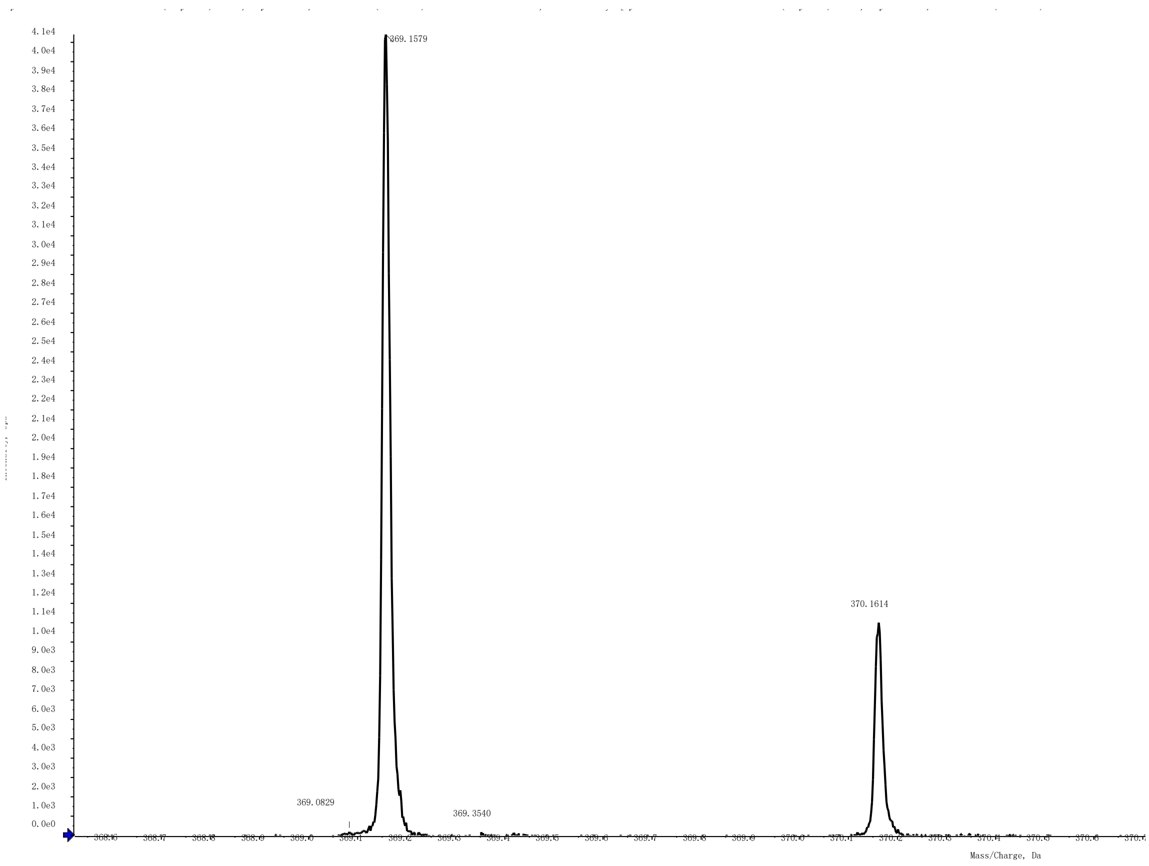

Figure S19 The HR-MS of compound **3**.

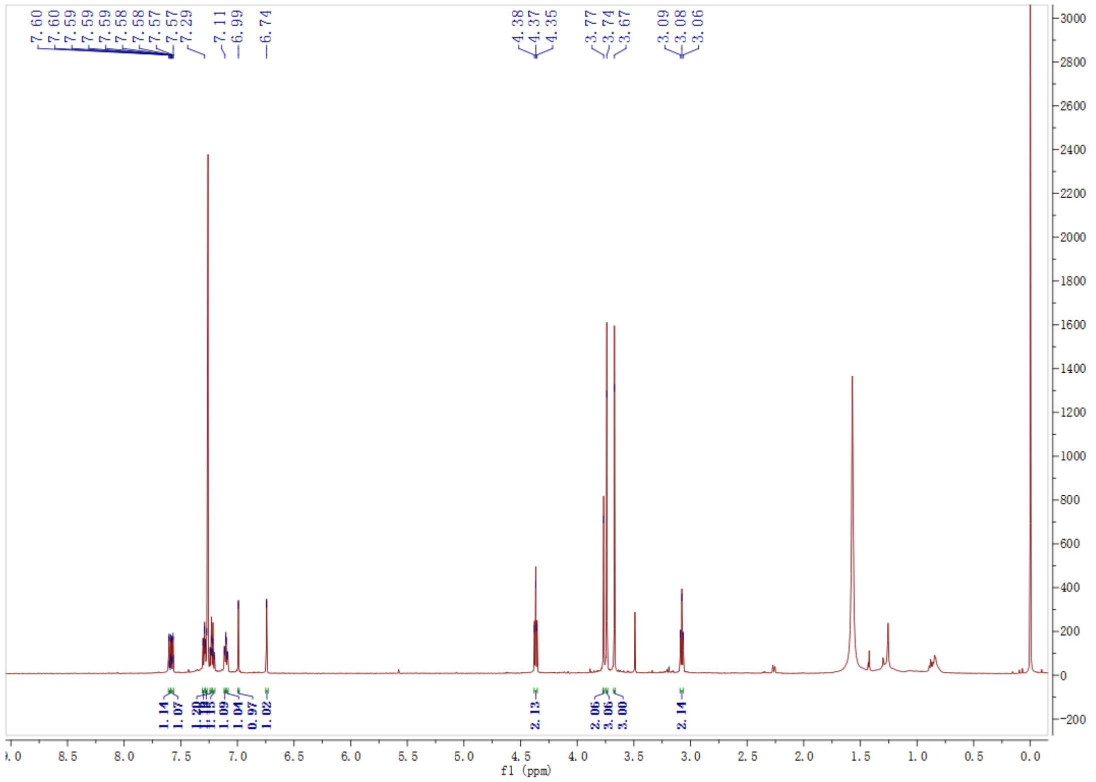

Figure S20 The ^{1}H NMR spectrum of compound **3** ($CDCl_3$, 600 MHz).

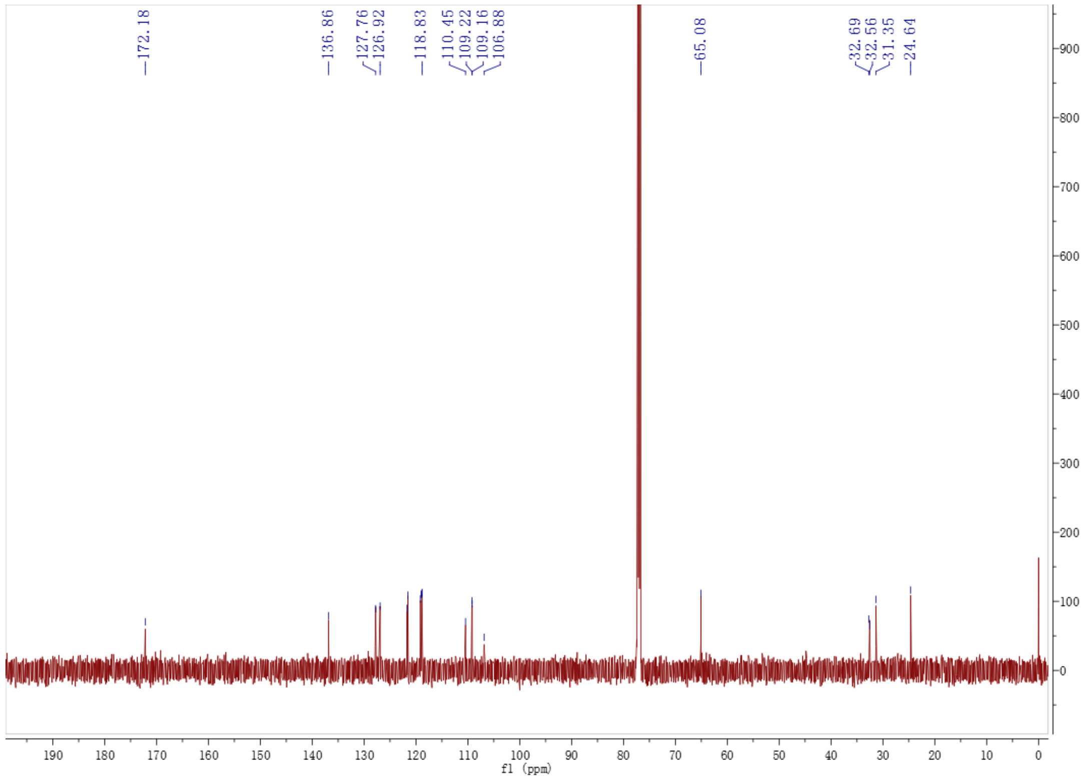

Figure S21 The ^{13}C NMR spectrum of compound **3** ($CDCl_3$, 151 MHz).

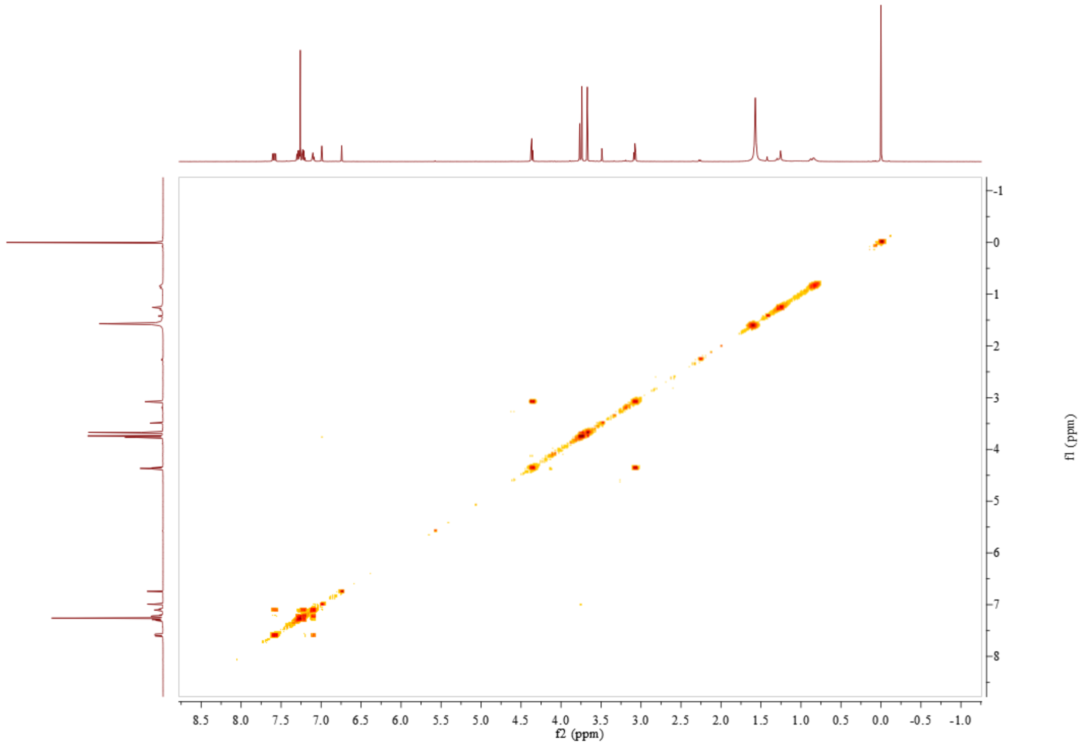

Figure S22 The ^{1}H-^{1}H COSY spectrum of compound **3** ($CDCl_3$, 600 MHz).

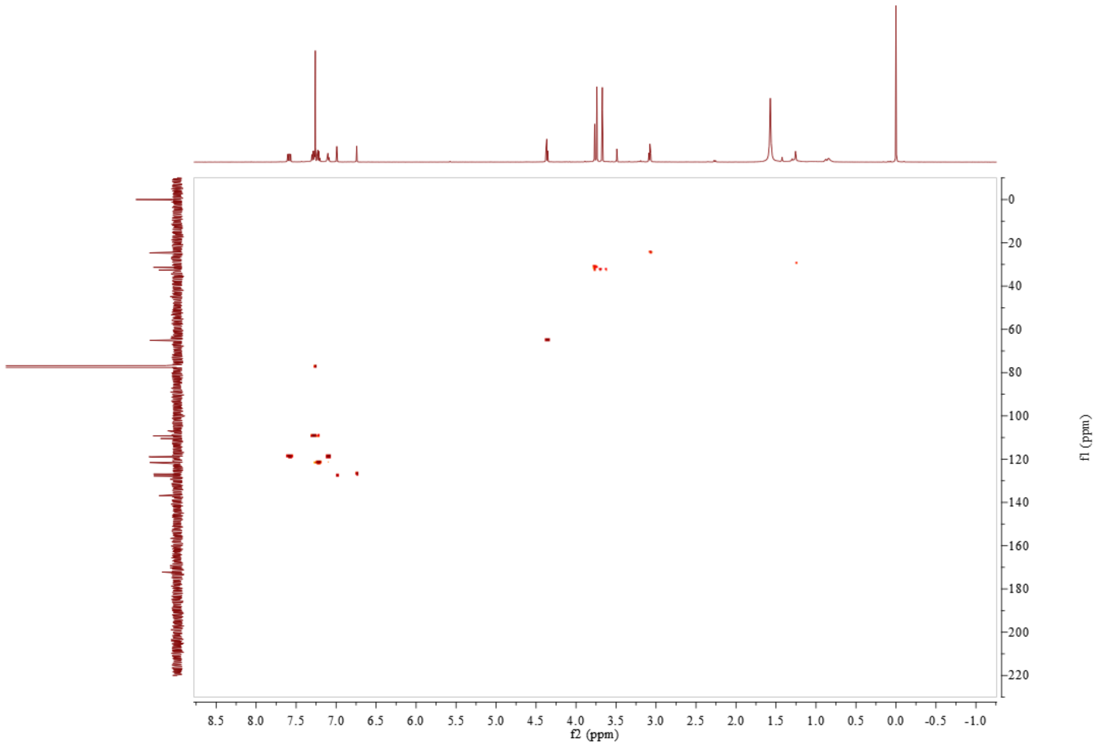

Figure S23 The HSQC spectrum of compound **3** ($CDCl_3$, 600 MHz).

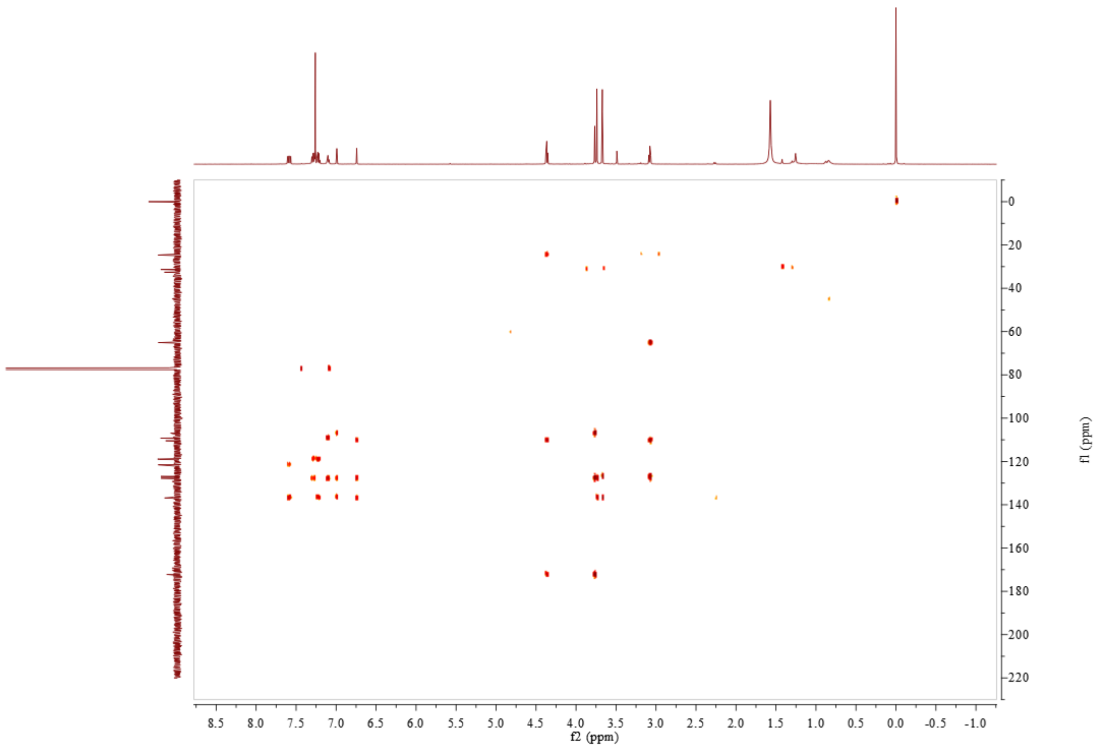

Figure S24 The HMBC spectrum of compound **3** ($CDCl_3$, 600 MHz).

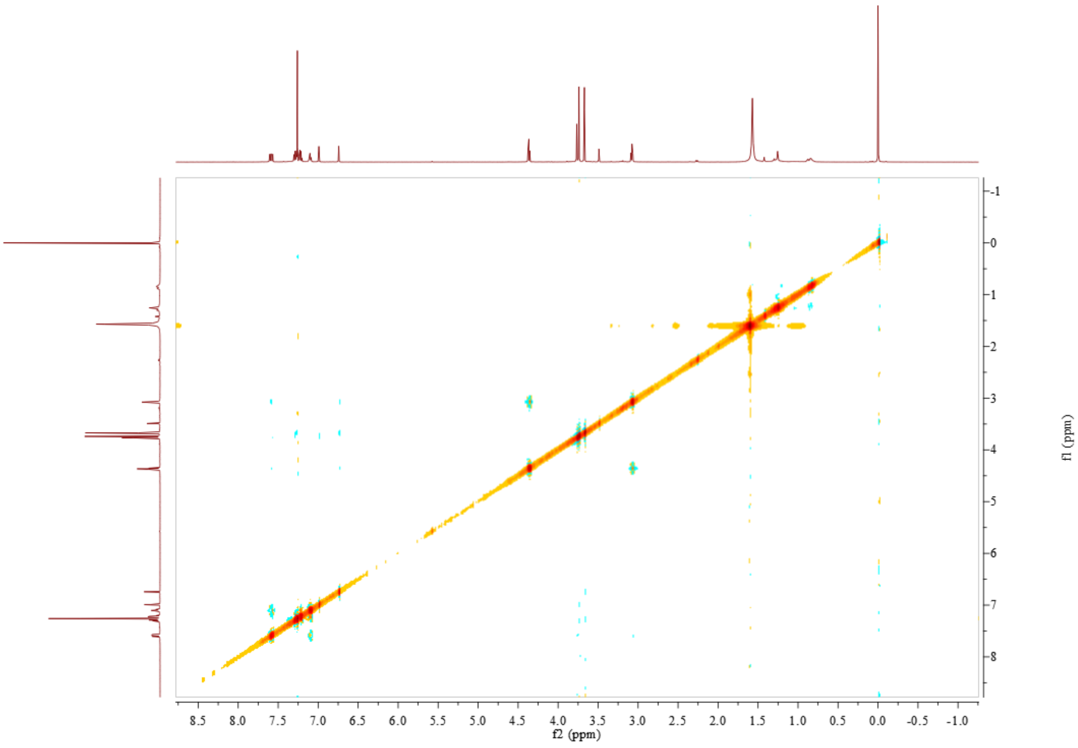

Figure S25 The NOESY spectrum of compound **3** ($CDCl_3$, 600 MHz).

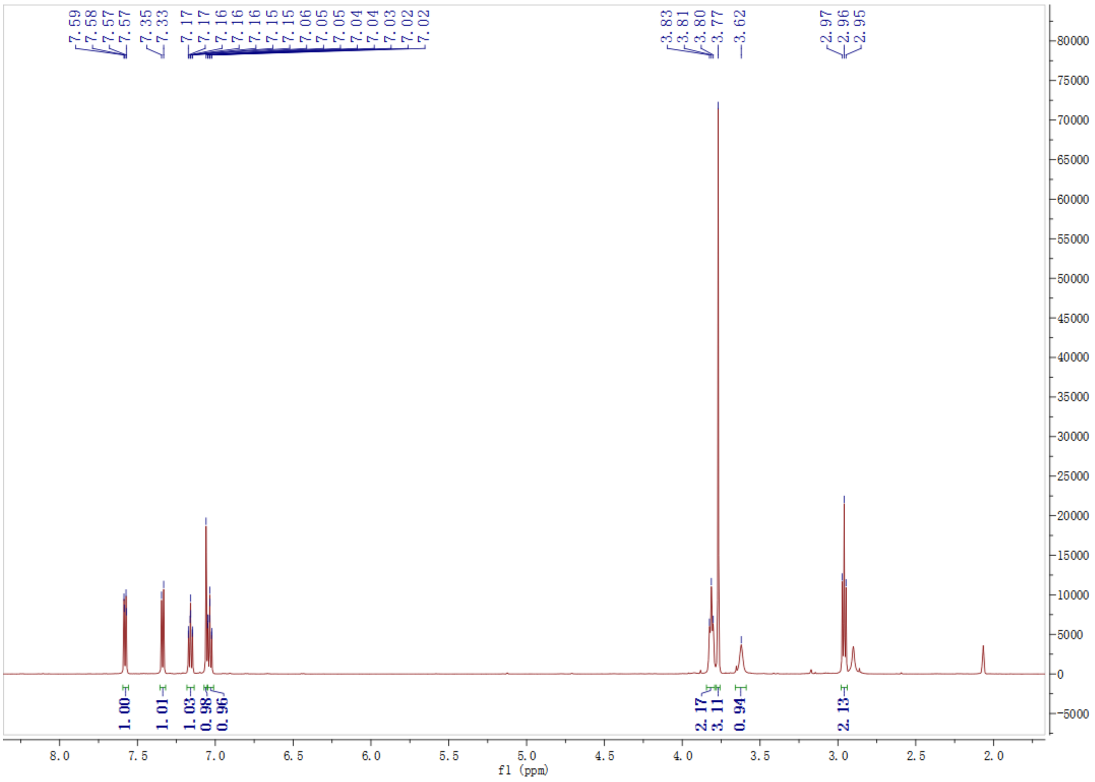

Figure S26 The ^{1}H NMR spectrum of compound **4** (Acetone-d_6, 600 MHz).

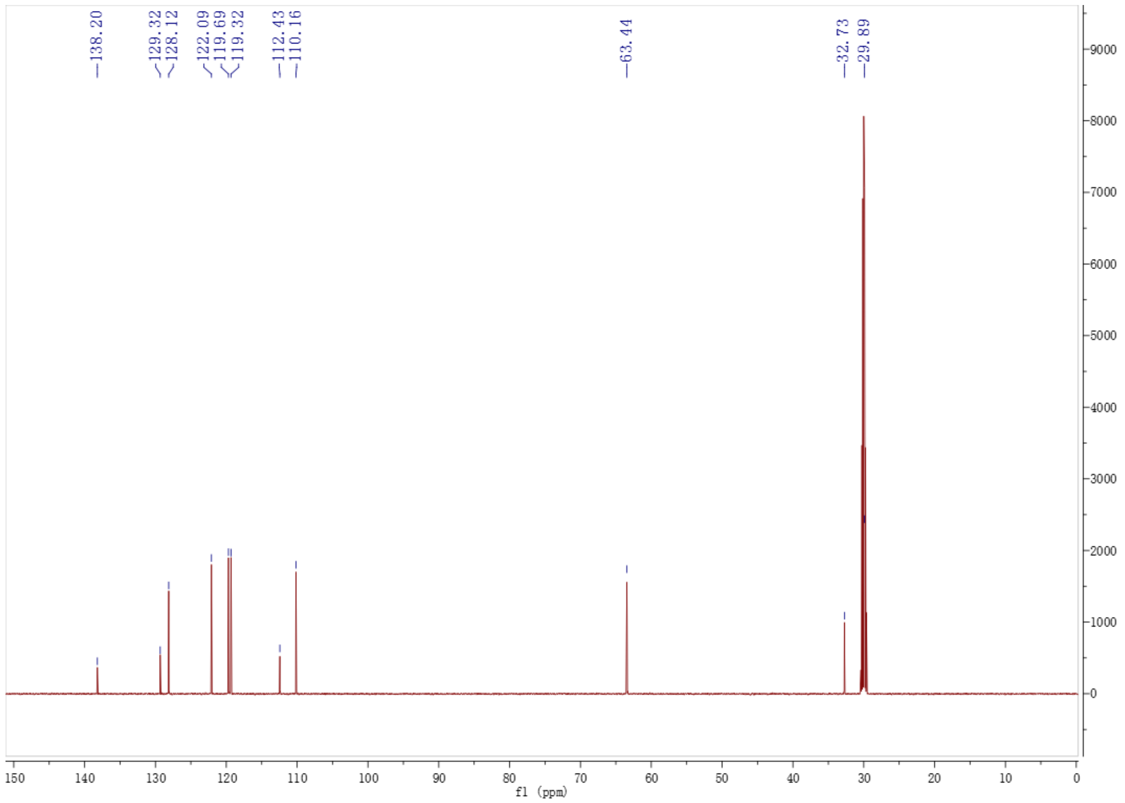

Figure S27 The ^{13}C NMR spectrum of compound **4** (Acetone-d_6, 151 MHz).

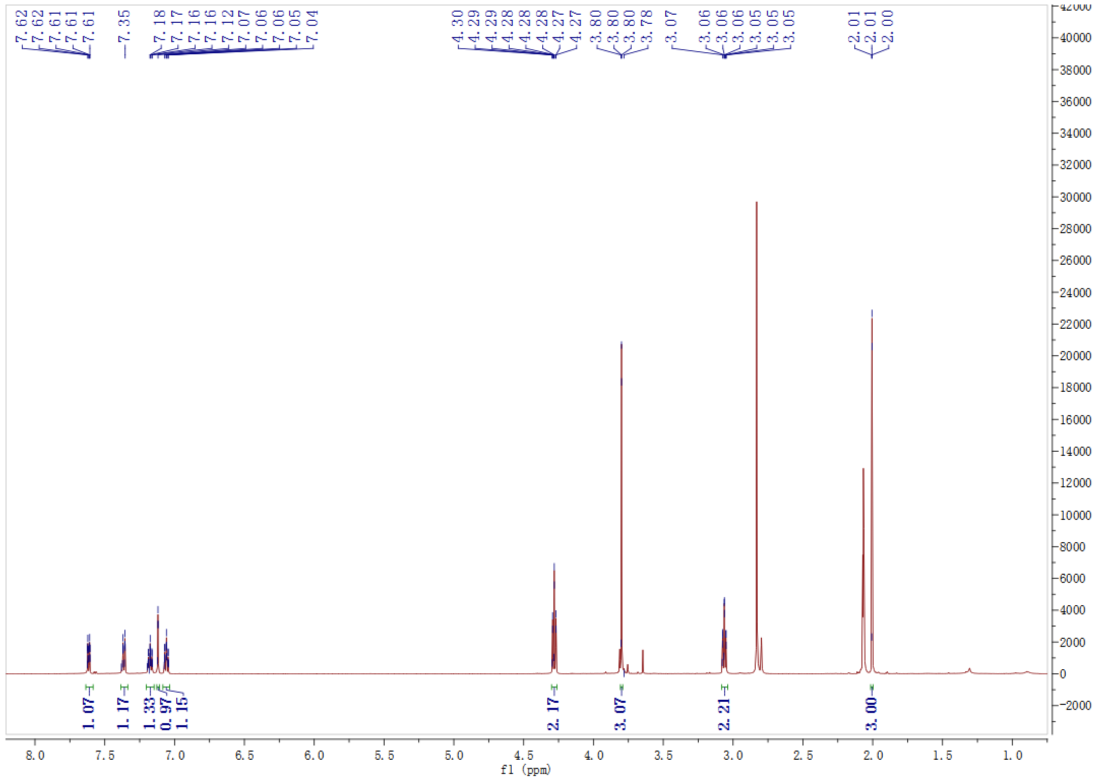

Figure S28 The ^{1}H NMR spectrum of compound **5** (Acetone-d_6, 600 MHz).

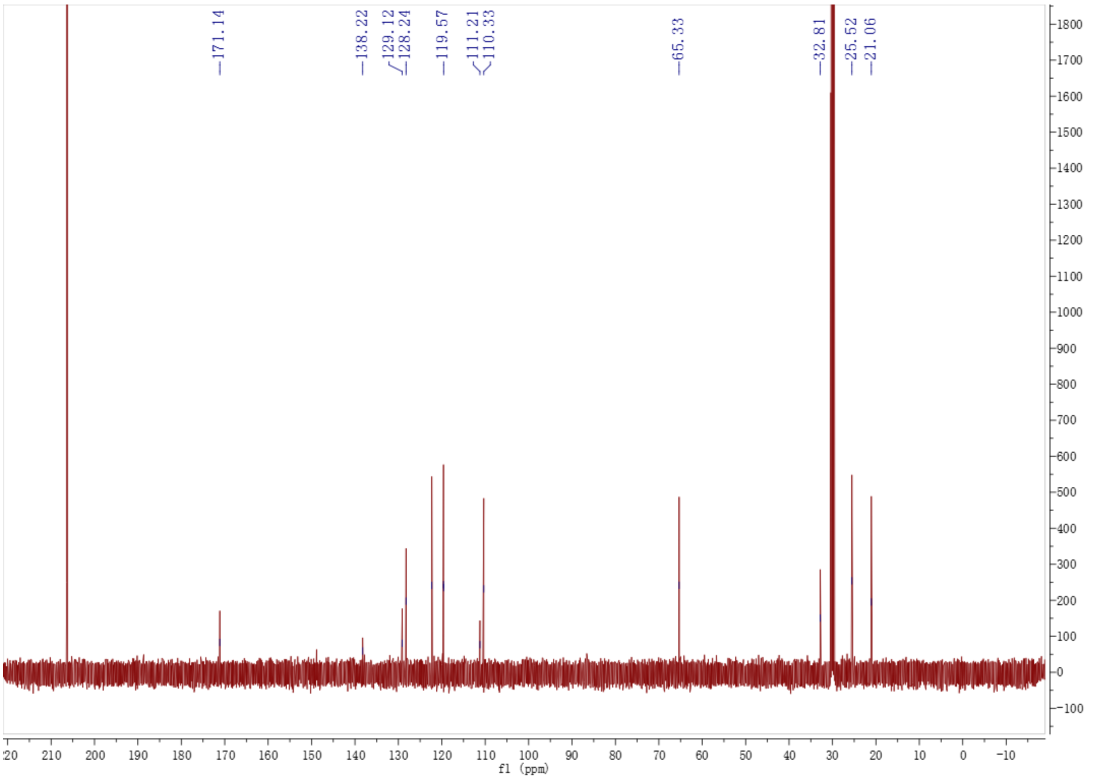

Figure S29 The ^{13}C NMR spectrum of compound **5** (Acetone-d_6, 151 MHz).

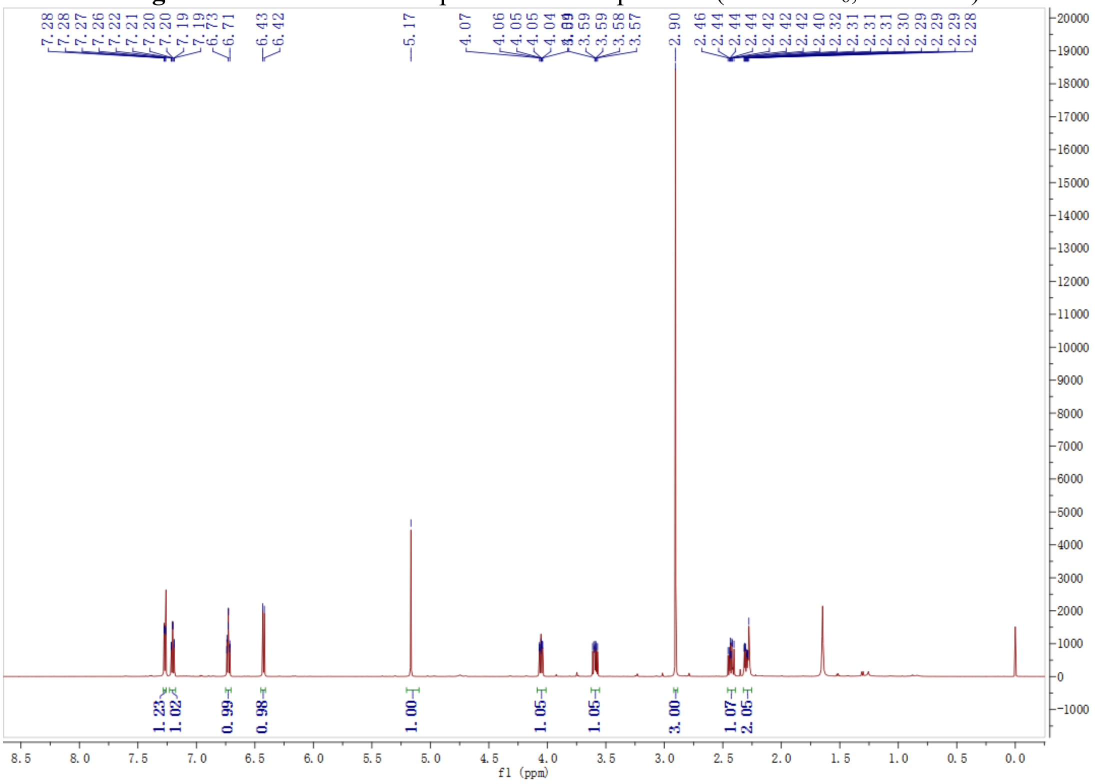

Figure S30 The ^{1}H NMR spectrum of compound **6** ($CDCl_3$, 600 MHz).

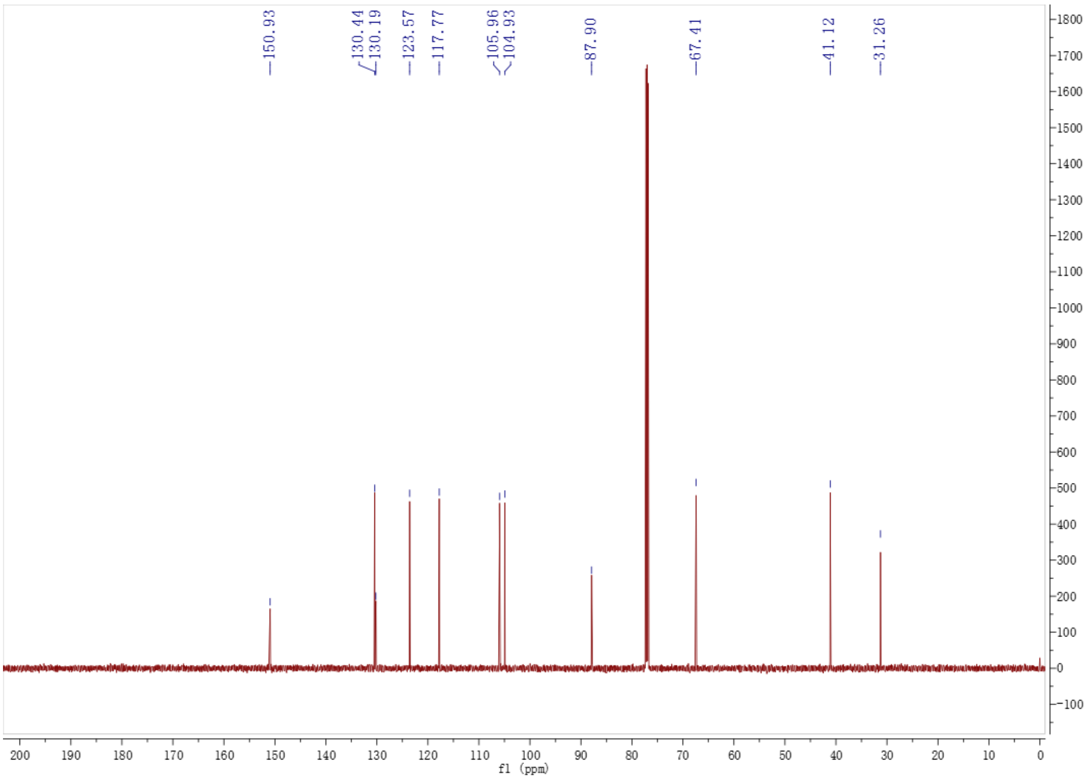

Figure S31 The ^{13}C NMR spectrum of compound **6** ($CDCl_3$, 151 MHz).

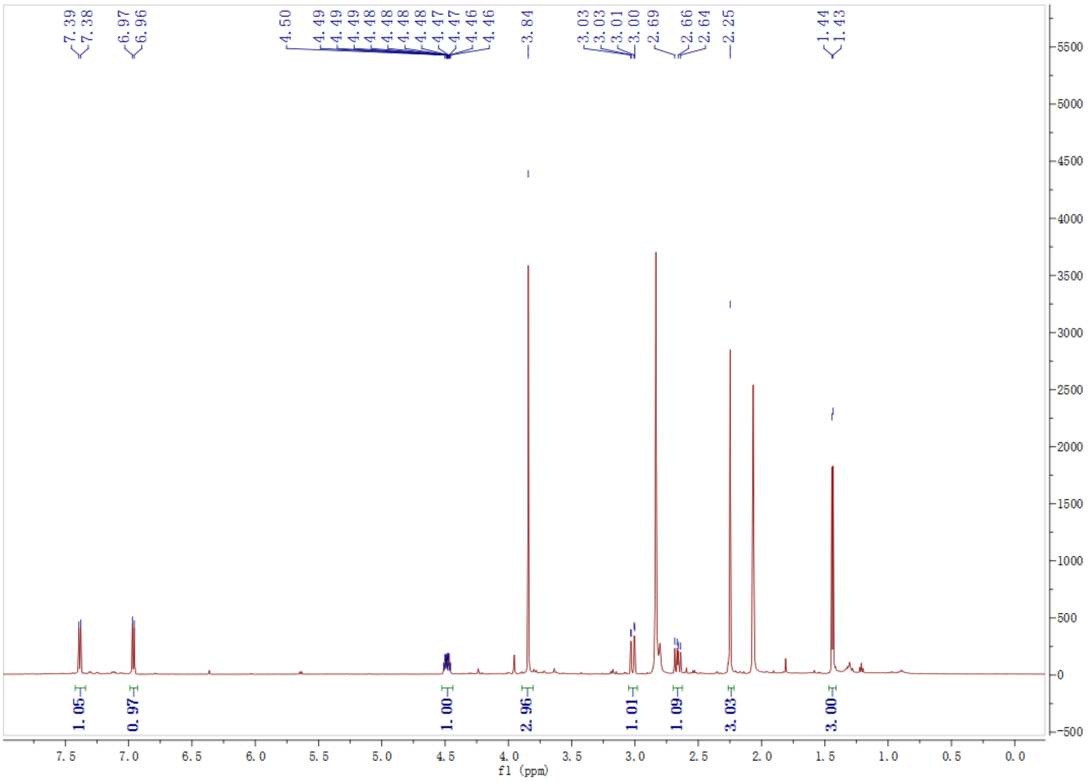

Figure S32 The ^{1}H NMR spectrum of compound **7** (Acetone-d_6, 600 MHz).

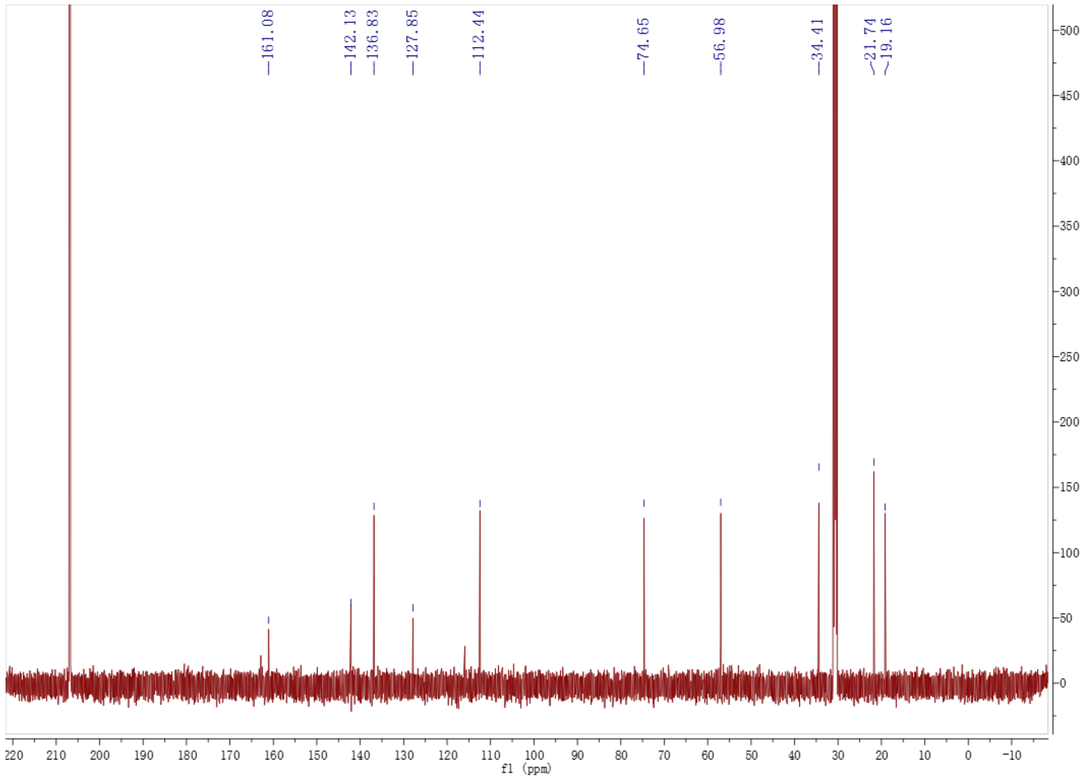

Figure S33 The ^{13}C NMR spectrum of compound **7** (Acetone-d_6, 151 MHz).

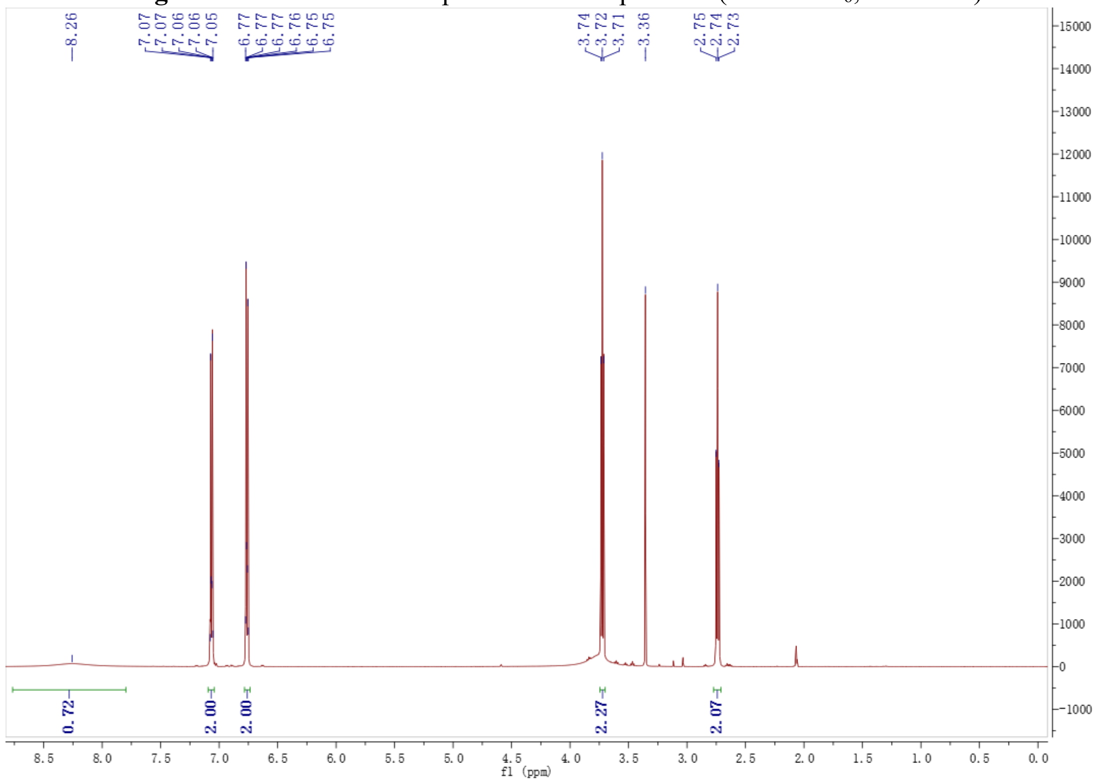

Figure S34 The ^{1}H NMR spectrum of compound **8** (Acetone-d_6, 600 MHz).

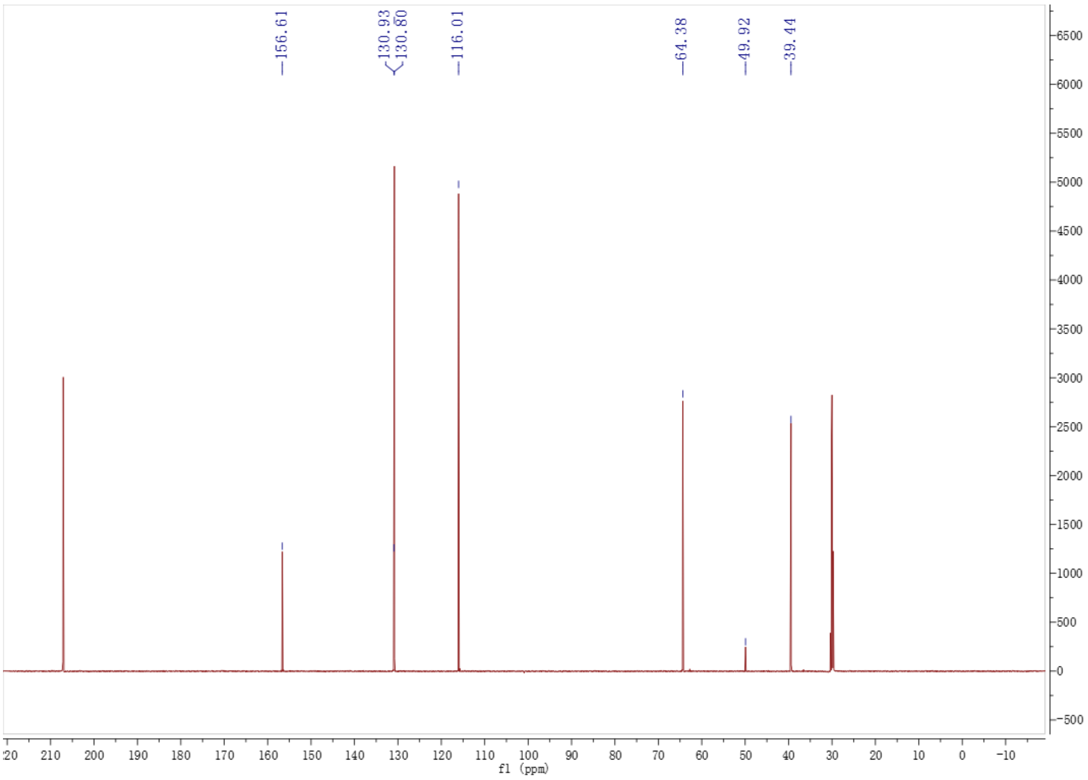

Figure S35 The ^{13}C NMR spectrum of compound **8** (Acetone-d_6, 151 MHz).

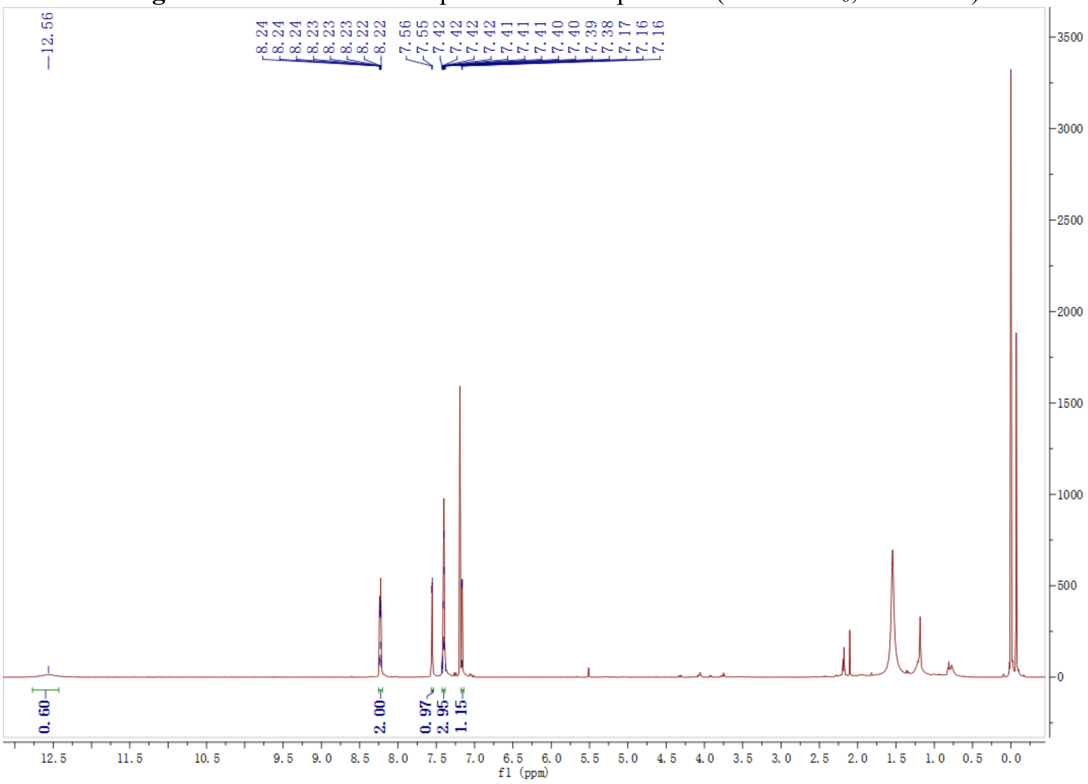

Figure S36 The ^{1}H NMR spectrum of compound **9** ($CDCl_3$, 600 MHz).

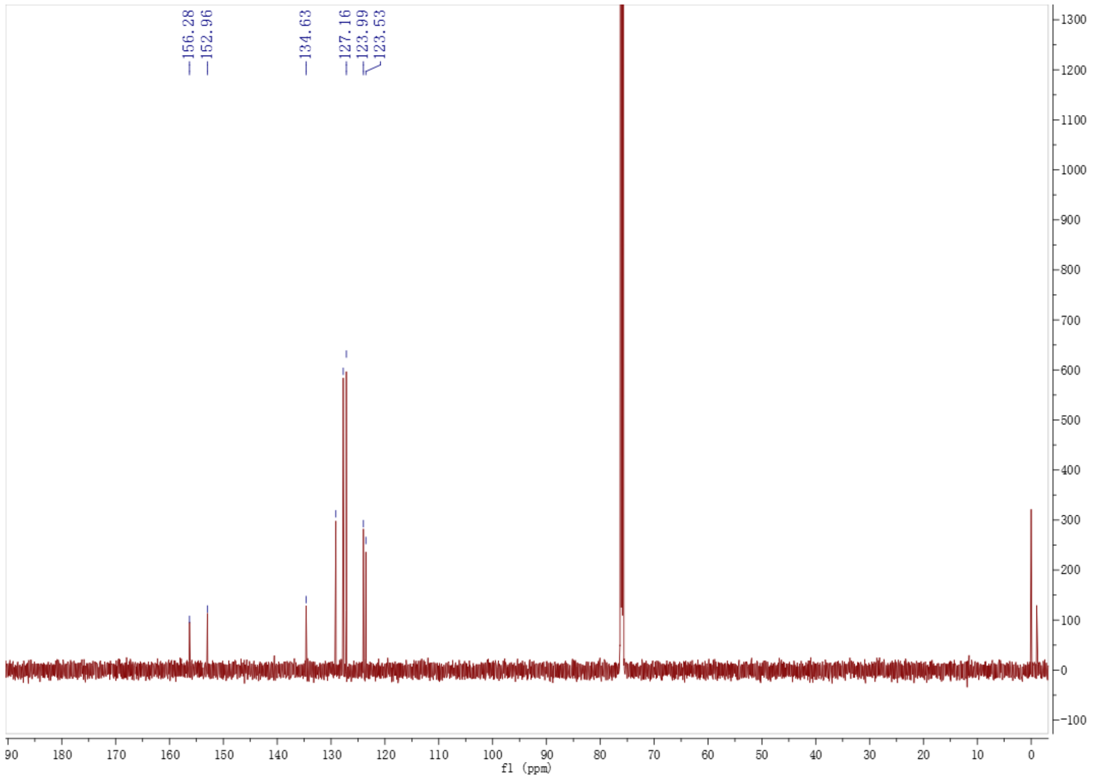

Figure S37 The ^{13}C NMR spectrum of compound **9** ($CDCl_3$, 151 MHz).
